# Tissue Mimetic Membranes for Healing Augmentation of Tendon–Bone Interface in Rotator Cuff Repair

**DOI:** 10.1002/adma.202407358

**Published:** 2025-01-31

**Authors:** Yuwei Zhu, Bingyang Dai, Shian Zhang, Jun Liu, Shunxiang Xu, Weiyang Liu, Xin Chen, Haozhi Zhang, Quan Li, Florence Ou‐Suet Pang, Weiguo Li, Chunyi Wen, Ling Qin, Jiankun Xu, To Ngai

**Affiliations:** ^1^ Department of Chemistry The Chinese University of Hong Kong Shatin, N. T. Hong Kong 999077 China; ^2^ Musculoskeletal Research Laboratory Department of Orthopaedics and Traumatology and Innovative Orthopaedic Biomaterial and Drug Translational Research Laboratory of Li Ka Shing Institute of Health Faculty of Medicine The Chinese University of Hong Kong Shatin Hong Kong 999077 China; ^3^ Department of Biomedical Engineering The Hong Kong Polytechnic University Hong Kong 999077 China; ^4^ The Hong Kong Polytechnic University Shenzhen Research Institute Shenzhen 518057 China; ^5^ Department of Physics The Chinese University of Hong Kong Shatin, N. T. Hong Kong 999077 China; ^6^ Department of Orthopaedic and Traumatology United Christian Hospital Kwun Tong Hong Kong 999077 China

**Keywords:** fibrocartilaginous interface regeneration, rotator cuff repair, tendon–bone healing, tissue mimetic membrane

## Abstract

The globally prevalent rotator cuff tear has a high re‐rupture rate, attributing to the failure to reproduce the interfacial fibrocartilaginous enthesis. Herein, a hierarchically organized membrane is developed that mimics the heterogeneous anatomy and properties of the natural enthesis and finely facilitates the reconstruction of tendon–bone interface. A biphasic membrane consisting of a microporous layer and a mineralized fibrous layer is constructed through the non‐solvent induced phase separation (NIPS) strategy followed by a co‐axial electrospinning procedure. Cationic kartogenin (KGN)‐conjugated nanogel (nGel‐KGN) and osteo‐promotive struvite are incorporated within the membranes in a region‐specific manner. During in vivo repair, the nGel‐KGN‐functionalized microporous layer is adjacent to the tendon which intends to suppress scar tissue formation at the lesion and simultaneously heightens chondrogenesis. Meanwhile, the struvite‐containing fibrous layer covers the tubercula minus to enhance stem cell aggregation and bony ingrowth. Such tissue‐specific features and spatiotemporal release behaviors contribute to effective guidance of specific defect‐healing events at the transitional region, further leading to the remarkably promoted regenerative outcome in terms of the fibrocartilaginous tissue formation, collagen fiber alignment, and optimized functional motion of rotator cuff. These findings render a novel biomimetic membrane as a promising material for clinical rotator cuff repair.

## Graphical abstract

Here, a tissue‐mimetic membrane for functional tendon–bone interface regeneration is fabricated. During the implantation, a biomimicry and inductive microenvironment is created by the region‐specific configuration and spatiotemporal release of chondroinductive kartogenin‐conjugated nanogel (nGel‐KGN) and osteoinductive struvite. The In Vitro and in vivo findings validate the prominent regenerative efficacy in rotator cuff repair.

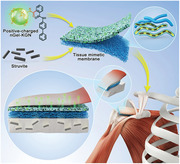

## 1 Introduction

Rotator cuff tear is one of the most common musculoskeletal disorders with a high prevalence (∼34%), which places a huge burden on both the healthcare and economic‐societal systems. Rotator cuff tears often occur at the tendon–bone insertion (i.e., enthesis) site, resulting in substantial pain and impairment of daily activities. Surgical repair is usually required to restore shoulder function in clinics, where more than 1.1 million/year rotator cuff tendon surgeries are performed globally.^[^ ^1^ ^]^ However, despite remarkable improvements in surgical techniques over the past decades, the rate of re‐tears after surgical intervention continues to range between 20% and 94%.^[^ ^2^, ^3^ ^]^ Such a high re‐rupture rate is closely related to the formation of highly disorganized fibrovascular scar tissue with an inferior biomechanical performance at the tendon–bone interface, rather than the regeneration of a gradient fibrocartilaginous transition.^[^ ^4^, ^5^ ^]^ Hence, there is an urgent clinical demand for tissue engineering strategies to reestablish the tendon‐to‐bone attachment close to the native level.

To this end, interface tissue engineering research into growth factors, stem cells and biological scaffolds has developed rapidly in recent years, with the biological‐scaffold‐mediated regenerative strategies in particular receiving immense attention.^[^ ^6^, ^7^, ^8^ ^]^ In clinical applications, the biological scaffolds are implemented in the forms of a film interposed between tendon and bone, or a patch bridging a massive defect to offer structural support, as well as a biomimicry microenvironment.^[^ ^9^ ^]^ The enthesis is a transitional region displaying gradients in structural, compositional, biofunctional, and biomechanical properties.^[^ ^10^ ^]^ Given the highly heterogeneous nature, dual‐phase/multi‐phase scaffolds show unparalleled superiority to the single‐phase ones for interface regeneration, where each phase is designed with different compositions and topographies to recapitulate the region‐specific properties of the natural enthesis.^[^ ^4^ ^]^ Numerous nanomaterials and nanofabrication technologies have been investigated to achieve a highly precisive simulation of the interface tissues and further facilitate tendon–bone healing.^[^ ^8^ ^]^ For instance, bioceramics, like nano‐sized hydroxyapatite (nHAp), are incorporated into a hierarchical scaffold to mimic the mineralized gradient from tendon to bone.^[^ ^2^ ^]^ Through electrospinning, the micro‐/nano‐structures of a scaffold can be tailored to reproduce the architecture of native tissue, as well as provide topographical cues to guide cellular behaviors and spur tissue formation.^[^ ^11^, ^12^, ^13^ ^]^ Nevertheless, though there is an increasing number of biological scaffolds reported with elaborate structures and advanced biomimetic properties, their application in vivo remains limited. Moreover, few studies have simultaneously addressed the problem of stem cells differentiating into cartilage and bone lineages at the transitional defect area, which are key points for augmenting the healing of tendon–bone interface.^[^ ^1^, ^9^, ^14^ ^]^

**Scheme 1:**
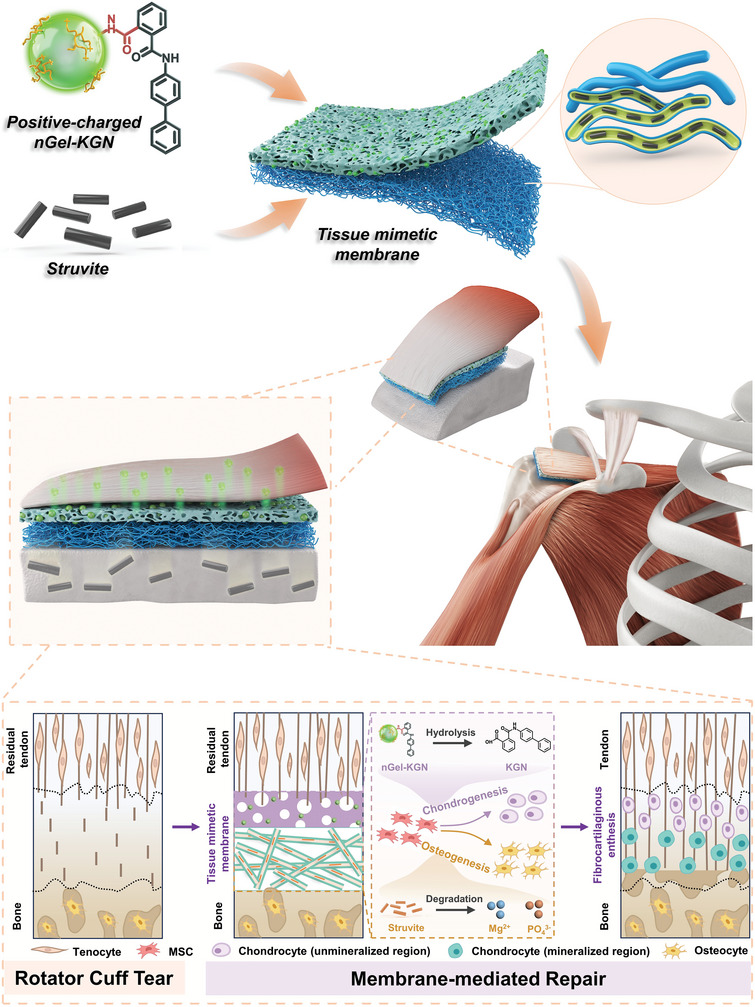
Schematic illustration of the biphasic structure of the tissue mimetic membrane and its application in a rotator cuff tear model for healing augmentation of tendon–bone interface. The biphasic membrane generates a bi‐lineage bioinducible effect as well as offers a supportive tissue mimetic platform, consequently promoting the interface tissue regeneration and tendon‐to‐bone reattachment.

In the present study, we aimed to produce a tissue mimetic membrane to generate an *in‐situ* co‐delivery of both osteo‐ and chondro‐promotive agents for functional tendon–bone repair. Kartogenin (KGN) is a well‐known heterocyclic druglike molecule that can promote selective differentiation of multipotent mesenchymal stem cells (MSCs) into the chondrogenic lineage and has recently been demonstrated to be capable of driving cartilage tissue formation at the injured tendon–bone junctions.^[^ ^15^ ^]^ Herein, the KGN‐conjugated poly (N‐isopropylmetharylamide) (PNIPMAM) nanogel (nGel) particle (nGel‐KGN) with weak positive surface charge was created for the first time and successfully incorporated into a microporous matrix for boosting chondrogenesis, showing more sustained and prolonged release pattern, as well as the optimized drug delivery efficiency in a restricted environment (rotator cuff), compared to the previous studies.^[^ ^15^, ^16^ ^]^ Though magnesium (Mg)‐containing bioceramics have been intensively investigated for bone regeneration, their effectiveness in augmenting tendon–bone healing remains unclear.^[^ ^1^, ^9^, ^17^, ^18^ ^]^ We pioneered to use the self‐synthesized osteo‐promotive struvite nanowires to construct the sharp mineral gradient mimicking the fibrocartilaginous transition zone, as well as spur osteogenesis at the defective interface. Consequently, a biphasic tissue mimetic membrane was fabricated through a casting procedure involving non‐solvent induced phase separation (casting‐NIPS) followed by co‐axial electrospinning, which is composed of a nGel‐KGN‐laden polycaprolactone (PCL)‐gelatin A (GelA) microporous layer and a mineralized PCL‐GelA core‐shell nanofibrous layer with struvite encapsulated in the PCL core. The as‐fabricated membrane was implanted between tendon and bone in the rat rotator cuff tear model, where the upper nGel‐KGN‐laden microporous layer simulated the interfacial unmineralized region whilst the lower struvite‐containing fibrous layer mimicking the mineralized region. In addition to being an *in‐situ* vehicle tuning the spatiotemporal release of nGel‐KGN and struvite to construct an inductive microenvironment, this tissue mimetic membrane also provided region‐specific compositional (i.e., mineralized gradient) and topographical cues to guide the healing process (e.g., specific tissue ingrowth, stem cell aggregation, and differentiation, etc.) (**Scheme** **1**). In Vitro and in vivo observations revealed the promotive effect of this tissue mimetic membrane and the beneficial role of the co‐release of nGel‐KGN/struvite on augmenting tendon–bone repair, implying the promising clinical curative efficacy in interface tissue repair.

## 2 Results

### 2.1 Synthesis and Characterization of the Positive‐Charged nGel‐KGN Particles

**Figure 1:**
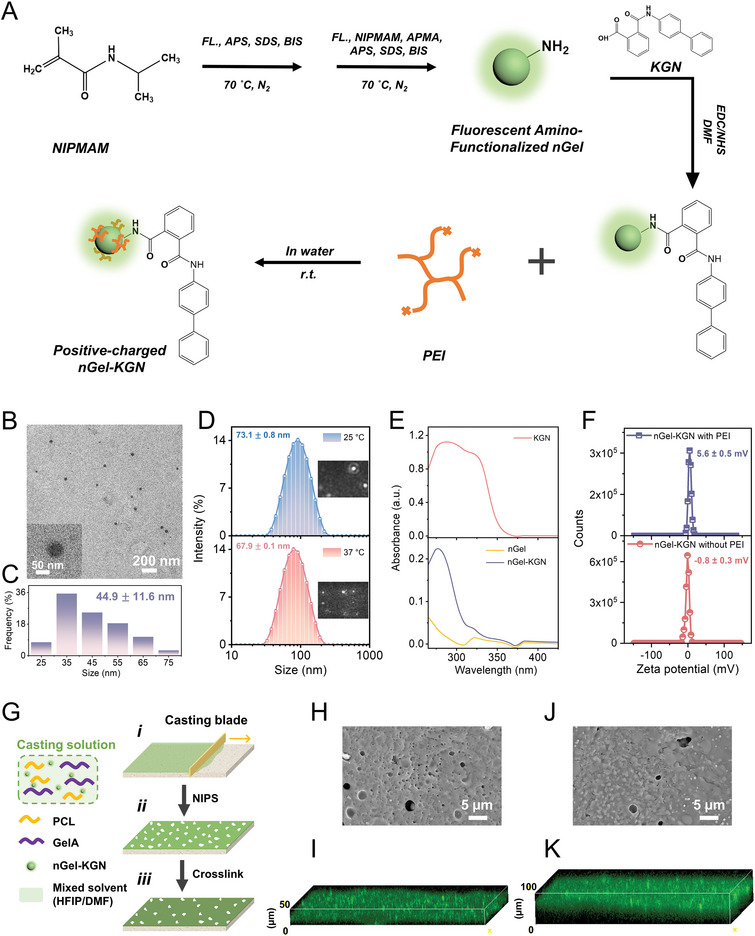
Synthesis of the positive‐charged kartogenin‐conjugated nanogel (nGel‐KGN) particles and fabrication of the nGel‐KGN‐laden microporous layer. A) Synthesis route of the positive‐charged nGel‐KGN particles. B) TEM images of the synthesized nGel‐KGN particles. C) Size distribution of the synthesized nGel‐KGN particles at dry state. D) Hydrodynamic size distribution of the synthesized nGel‐KGN particles in 2.5 mM HEPES buffer solution at 25 and 37 °C. Insets: the video frames of the nGel‐KGN particles in 2.5 mM HEPES buffer solution at 25 and 37 °C, obtained from nanoparticle tracking analysis (NTA) measurements. E) UV–vis spectra of KGN, nGel, and nGel‐KGN as dissolved in dimethyl sulfoxide (DMSO). F) Zeta potential of the nGel‐KGN particles with and without PEI in 2.5 mM HEPES buffer solution. G) Fabrication of the nGel‐KGN‐incorporated microporous layer through casting combined with non‐solvent induced phase separation technique. H) SEM image and I) 3D fluorescent image of the 500 µm‐casting/5% nGel‐KGN layer. J) SEM image and K) 3D fluorescent image of the 1000 µm‐casting/5% nGel‐KGN layer.

Drug conjugation to polymeric carriers is regarded effectively to enhance solubility and permeability, as well as optimize the pharmacokinetics in the physiological environment.^[^ ^19^ ^]^ Aqueous microgel/nanogel particles are extensively investigated as vectors to deliver a wide range of bioactive, owing to their prominent loading capacity and stimuli‐responsiveness.^[^ ^16^, ^20^ ^]^ Herein, amino‐functionalized PNIPMAM nGel particles were synthesized for KGN (with carboxyl group) conjugation through the EDC/NHS‐activated amine‐acid coupling, which were further coated with poly (ethyleneimine) (PEI) polymer chains to generate the cationic nGel‐KGN conjugate (**Figure** **1**A). The resulting nGel‐KGN particles displayed spherical with an average size of 44.9 ± 11.6 nm at the dry state (Figure 1B,C). Nanogels/microgels are a class of crosslinked colloidal particles that can swell by absorbing the solvent,^[^ ^16^ ^]^ and a significant increase of the hydrodynamic size (73.1 ± 0.8 nm at 25 °C) was observed in the nGel‐KGN particles after swollen by the HEPES buffer (Figure 1D). Considering the lower critical solution temperature (LCST) of the thermoresponsive PNIPMAM polymer is ∼44 °C,^[^ ^20^ ^]^ the nGel‐KGN particles remained highly swollen when the temperature increased to 37 °C with negligible shrinkage (67.9 ± 0.1 nm), implying the satisfactory phase stability in the body (Figure 1D and Movies S1, S2, Supporting Information). Further, the valid conjugation of the KGN molecules onto the nGel surface was determined by the strong UV absorption at ∼277 nm (Figure 1E) and the existence of the major peaks of benzene rings in KGN at 7.3–7.9 ppm as revealed by the ^1^H NMR spectra (Figure S1A, Supporting Information).^[^ ^21^, ^22^ ^]^ The binding degree of KGN was determined as 4.06 × 10^−5^ mol KGN/g nGel (Equation 1 and Figure S2, Supporting Information). Through the physical adsorption driven by the polymer chain entanglement, the nGel‐KGN particles (near neutral) were coated with a small amount of cationic PEI polymers and gained the weak positive charge (5.6 ± 0.5 mV) (Figure 1F), to promote the introduction of drugs into cells.^[^ ^16^ ^]^

### 2.2 Fabrication and Characterization of the Biphasic Tissue Mimetic Membranes

The well‐synthesized cationic nGel‐KGN was readily incorporated within a microporous polymer composite layer as the chondro‐inductive agents through the casting‐NIPS method (Figure 1G i,ii). Considering the effective dosage of KGN and the safe (non‐cytotoxic) concentration range of PNIPMAN nanogels, the initial screening for the loading concentration of nGel‐KGN was set as 0%, 5%, and 10%, based on the literature.^[^ ^23^, ^24^, ^25^ ^]^ Given a high particle concentration (i.e., 10%) would induce more interface defects of the membranes due to the increased particle agglomeration (Figure S3, Supporting Information),^[^ ^26^, ^27^ ^]^ and may deteriorate the mechanical performance during the implantation,^[^ ^28^ ^]^ a 5% concentration (containing 0.065% KGN) was chosen as the final loading level to prepare the microporous layer. The small portion of the non‐solvent (i.e., DMF) drove the liquid‐liquid phase separation, where the polymer‐rich phase formed as the nuclei first and gradually grew into secondary particles. The subsequent coagulation of secondary particles contributed to the highly interconnected porous structure in the resulting dry membrane (Figure S1D, Supporting Information).^[^ ^29^ ^]^ After crosslinking, the surface porosity decreased, leading to the formation of the targeting selective‐permeable microporous layer with homogenously dispersed nGel‐KGN particles (Figure 1G iii, H–K), which is expected to exclude the scar tissue interference at early healing stage and deliver chondro‐inductive cues in vivo.

**Figure 2:**
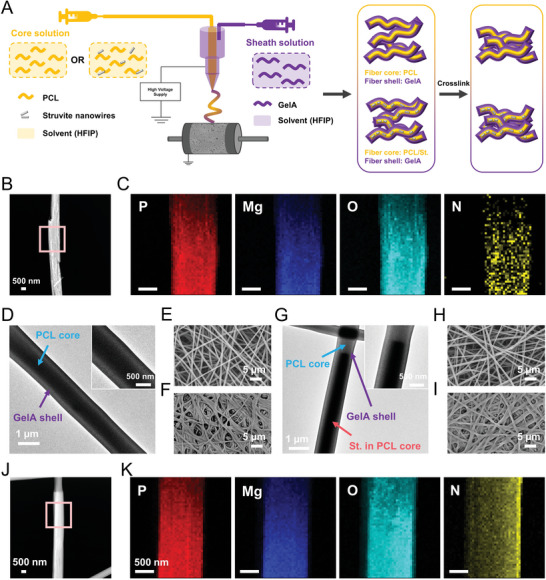
Fabrication of core‐shell electrospun fibrous layer. A) Schematic illustration of producing core‐shell fibers through co‐axial electrospinning (ES) technique. The PCL‐GelA core‐shell fibers were directly electrospun onto the microporous layer to produce the second struvite (St.)‐incorporated fibrous layer, and the targeting biphasic tissue mimetic membrane was obtained after a crosslinking procedure by EDC/NHS. B) High‐angle annular dark‐field image of St. nanowires and C) the corresponding EDS maps of the compositional elements P, Mg, O, and N. D) TEM image of the PCL‐GelA core‐shell fiber. E,F) SEM images of the PCL‐GelA core‐shell fibrous membrane (E) before and (F) after crosslinking. G) TEM image of the PCL/St.‐GelA core‐shell fiber. H,I) SEM images of the PCL/St.‐GelA core‐shell fibrous membrane (H) before and (I) after crosslinking. J) High‐angle annular dark‐field image of a single PCL/St.‐GelA core‐shell fiber and K) the corresponding EDS maps of the compositional elements P, Mg, O, and N.

Subsequently, the struvite (St.)‐encapsulated core‐shell nanofibers were directly electrospun onto the microporous layer to produce the second fibrous layer, and a crosslinking procedure was performed via the coupling reaction of amine and carboxyl groups in GelA polymer to enhance the cohesion of the two layers, as well as promote the mechanical performance of this biphasic tissue mimetic membrane (**Figure** **2**A).^[^ ^17^ ^]^ The struvite nanowires with nano‐sized diameter and high phase purity were successfully synthesized through the controlled crystallization strategy (Figure 2B; Figure S1E,F, Supporting Information). The major compositional elements of phosphorous (P), Mg, oxygen (O), and nitrogen (N) are evenly distributed throughout the struvite nanowires as evidenced by the EDS mapping (Figure 2C). These nanowire‐like particles were then encapsulated into the core‐shell polymer fibers via co‐axial electrospinning (Figure 2A). The core‐shell structures of the PCL‐GelA and PCL/St.‐GelA nanofibers were visualized by TEM imaging (Figure 2D,G). The PCL core was displayed as the dark compartment inside the composite, while the GelA shell was grey, resembling a thin coating of a single PCL fiber (Figure 2D). The struvite nanowire was embedded within the PCL core along the fiber orientation (Figure 2G,J), which was further verified by the strong signal of the characteristic compositional elements, Mg and P, in EDS analysis (Figure 2K). The SEM imaging showed the successful preparation of beads‐free nanofibrous layers laden without and with struvite nanowires (Figure 2E,H), and the packing density of the nanofibers remarkably increased after crosslinking (Figure 2F,I). This extracellular matrix (ECM)‐mimic core‐shell nanofibrous layer is expected to show prominent cell recruitment capacity during implantation and a controllable release of the osteo‐promotive cues in situ.

Consequently, three kinds of tissue mimetic membranes with different structures, thickness, and compositions were fabricated: 500 µm‐casting + ES (500C‐ES), 500 µm‐casting/5% nGel‐KGN + ES/4% St. (500C/nG‐ES/St), and 1000 µm‐casting/5% nGel‐KGN + ES/4% St. (1000C/nG‐ES/St) (Table S1, Figure S1G,J,K, Supporting Information). The cross‐sectional morphological analysis revealed the good cohesion of the two layers in three groups, demonstrating the well‐constructed biphasic structures (Figure S1H, Supporting Information). The characteristic absorption bands of PCL and GelA polymers without evident peak shift were observed in the FTIR spectra of all three membranes, and no new peaks appeared, suggesting that the presence of nGel‐KGN/struvite within the polymer matrix would not induce new compound formation (Figure S1I, Supporting Information).^[^ ^30^, ^31^ ^]^ Additionally, a similar thermal decomposition tendency was found in the three membranes, implying that the small‐amount nGel‐KGN/struvite incorporation had a neglectable effect on the thermal stability of the membranes (Figure S1L, Supporting Information). The thermal decomposition of the membranes experienced three consecutive stages – Stage 1: the loss of surface and bonded water of the GelA moieties (room temperature to 260 °C); Stage 2: the decomposition of GelA (260–360 °C); Stage 3: the degradation of PCL (360–430 °C).^[^ ^32^ ^]^

### 2.3 Surface Properties of the Tissue Mimetic Membranes

**Figure 3:**
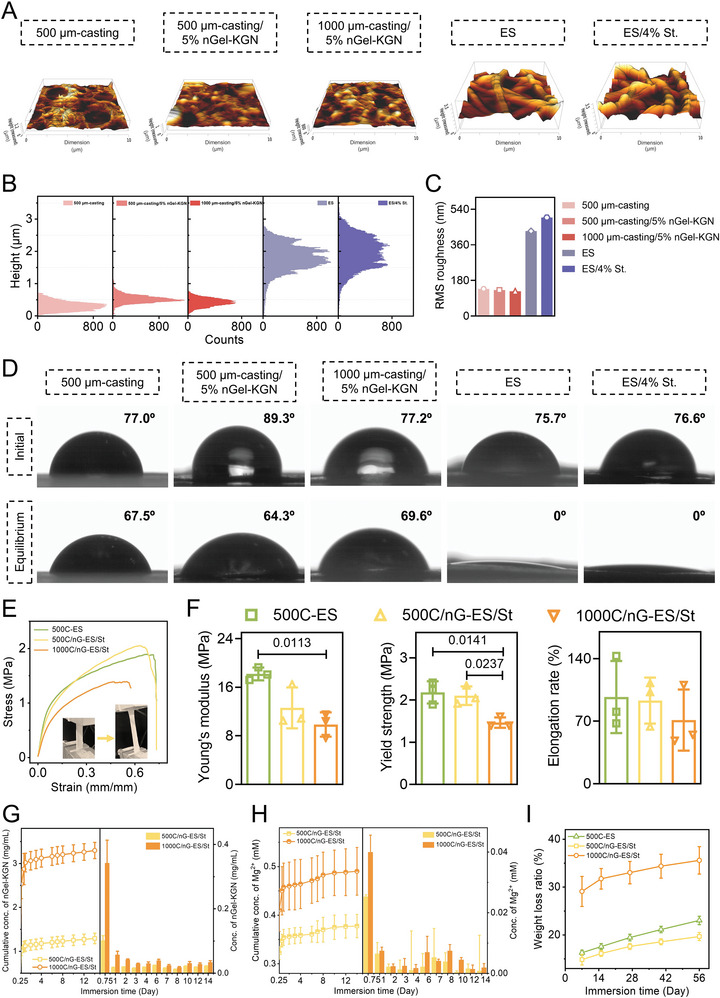
Characterizations of the fabricated tissue mimetic membranes – 500C‐ES, 500C/nG‐ES/St, and 1000C/nG‐ES/St. A) 3D AFM images, B) height histogram, and C) root mean square (RMS) roughness of different microporous and fibrous layers: 500 µm‐casting, 500 µm‐casting/5% nGel‐KGN, 1000 µm‐casting/5% nGel‐KGN, ES, ES/4% St. D) Representative optical images of water droplets on different membrane surfaces at initial and equilibrium states during the wetting process. The measured water contact angle (WCA) values were displayed at the upper right corner of each image. E) Representative strain‐stress curves of the three fabricated membranes in a wet state obtained from tensile tests. Insets: representative photos of the 500C/nG‐ES/St membrane during tensile test. F) Young's modulus, yield strength, and elongation rate of different membranes in a wet state were calculated from the strain‐stress curves (*n* = 3). G,H) The 14‐day release profile of (G) nGel‐KGN particles and (H) Mg^2+^ from the 500C/nG‐ES/St and 1000C/nG‐ES/St membranes in PBS solution (*n* = 3). I) The 56‐day degradation profiles of the three fabricated membranes in PBS solution (*n* = 3). The quantitative data are presented as the mean ± SD. One‐way ANOVA with *Tukey's post hoc* test (F) was used.

The surface roughness of as‐fabricated biphasic tissue mimetic membranes was examined using atomic force microscopy (AFM) (**Figure** **3**A; Figure S4A, Supporting Information). In general, the microporous layers exhibited a significantly smoother surface in comparison with the fibrous layers (Figure 3B,C). Besides, there were small variations among the different microporous and fibrous surfaces. During phase inversion, the embedded nGel‐KGN particles acted as the nucleating agents and suppressed the free growth of nuclei, leading to a denser surface and slightly decreased root mean square (RMS) roughness in 500 µm‐casting/5% nGel‐KGN layer compared to 500 µm‐casting layers (from 137.3 to 131.3 nm) (Figure 3B,C; Figure S1G, Supporting Information).^[^ ^33^, ^34^, ^35^ ^]^ Further reduced surface porosity and roughness were found in the 1000 µm‐casting/5% nGel‐KGN layer (125 nm) since the increased casting thickness can retard the polymer precipitation at the polymer‐solvent‐non‐solvent interface (i.e., membrane surface).^[^ ^36^ ^]^ Conversely, the introduction of struvite nanowires into the fibrous layer contributed to an elevated roughness from 429.6 to 498.3 nm (Figure 3B,C). It is assumed that in the crosslinking and subsequent drying process, polymer fibers may collapse to connect, while the hard struvite nanowires within the fiber core tend to keep the original shape, thus inducing a more uneven surface. Previous studies indicated that a smooth surface could promote fibroblast adhesion and collagen deposition, while a randomly fibrous surface would aid osteogenic differentiation of MSCs and osteoblast aggregation.^[^ ^11^, ^37^ ^]^ Therefore, by interposing between tendon and bone (Scheme 1), the tissue‐specific nano‐topographical features of these two surfaces (i.e., the direct interface) are expected to enable prominent tendon/bone–membrane integration simultaneously.

All the membrane surfaces showed high water‐affinity due to the presence of hydrophilic GelA polymer, which would be a beneficial factor for tissue regeneration (Figure 3D).^[^ ^17^, ^38^ ^]^ Upon contact with the surfaces, the water droplet was absorbed by the fibrous surface shortly (water contact angle, WCA = 0°), which was driven by the GelA fiber shell in the fibrous layer. As for the microporous surfaces, water absorption capacity was restrained by the hydrophobic PCL with the WCA lower than 70° at the equilibrium state.^[^ ^39^ ^]^ However, the introduction of nGel‐KGN and struvite to the specific layers did not show an effect.

### 2.4 Mechanical Performance of the Tissue Mimetic Membranes

Tendon‐to‐bone interface is an essential site to allow stress transfer, so appropriate mechanical strength of the biological scaffolds is vital regarding the interface regeneration.^[^ ^40^ ^]^ Considering the humid physiological microenvironment, we performed tensile tests to evaluate the mechanical performance of the as‐prepared membranes in a wet state to simulate the real application scenario (Figure 3E,F). No significant difference was found in the yield strength (*σ*) and Young's modulus (*E*) of 500C‐ES (*σ* = 2.18 ± 0.26 MPa, *E* = 18.19 ± 1.05 MPa) and 500C/nG‐ES/St (*σ* = 2.10 ± 0.22 MPa, *E* = 12.61 ± 3.37 MPa), suggesting that the nGel‐KGN/struvite incorporation had little influence on the mechanical properties of the dual‐phase membrane at the applied loading amount. The increase of the thickness of the nGel‐KGN‐laden microporous layer induced remarkably impaired mechanical behaviors in 1000C/Ng‐ES/St membrane (*σ* = 1.47 ± 0.12 MPa, *E* = 9.86 ± 1.99 MPa) (Figure 3F; Figure S1J,K, Supporting Information). As aforementioned, the microporous structure was formed by multiple particle coagulation, which would result in a relatively lower mechanical stability compared to the fibrous layer that was generated by the entanglement of myriads of polymer fibers (Figure S1D,G, Supporting Information).^[^ ^41^ ^]^ Hence, the increased proportion of the weaker microporous layer in the biphasic tissue mimetic membrane (1000C/nG‐ES/St) would deteriorate the mechanical performance. Satisfyingly, all three membranes showed comparable mechanical strength to the most reported electrospun films applied for rotator cuff repair.^[^ ^1^ ^]^ Besides, the high elongation rate of the three membranes demonstrated good deformability to ease the implantation procedure (Figure 3F).^[^ ^17^ ^]^

### 2.5 In Vitro Release and Degradation Profiles of the Tissue Mimetic Membranes

Owing to the good hydrophilicity (Figure 3D), the embedded nGel‐KGN and struvite could easily be transferred from the membrane matrix to the surrounding microenvironment during implantation, driven by the concentration gradient. Given the nGel‐KGN suspension in PBS buffer showed a linear fluorescence emission intensity at the concentration of 0.1–3.75 mg mL^−1^ (Figure S1B,C, Supporting Information), we monitored the nGel‐KGN release by measuring the fluorescence intensity of the soaking solution. The release tendency of nGel‐KGN was similar in 500C/nG‐ES/St and 1000C/nG‐ES/St, that a quick release occurred on the first day and then turned to be more controllable (Figure 3G). The increased thickness of nGel‐KGN‐laden microporous layer contributed to the higher loading amount of nGel‐KGN in 1000C/nG‐ES/St (Figure S1J, Table S1, Supporting Information), so the cumulative concentration of nGel‐KGN was significantly higher in 1000C/nG‐ES/St (∼3.29 mg mL^−1^) than that in 500C/nG‐ES/St (∼1.29 mg mL^−1^) after the 14‐day release. However, the difference in nGel‐KGN concentration level at certain time points became smaller from the 3^rd^ day between the two membranes (Figure 3G). To further determine the release behaviors of struvite, we measured the concentration of the major degradation products (i.e., Mg ions [Mg^2+^]), which also potentially play a key role in promoting osteogenesis.^[^ ^18^, ^42^ ^]^ A rapid increase of Mg^2+^ level appeared in both groups (500C/nG‐ES/St: ∼0.35 mM; 1000C/nG‐ES/St: ∼0.46 mMm) after 1‐day release, and then the release rate gradually slowed down (Figure 3H). The release levels of Mg^2+^ continued to be comparable in 500C/nG‐ES/St and 1000C/nG‐ES/St, corresponding to the same configuration of the struvite‐incorporated fibrous layer. Considering the In Vitro release conditions simulated the timely consumption of the agents in vivo, the release tendency in vivo was expected to be closely aligned with the In Vitro results, that a sustained and prolonged delivery of the two therapeutic agents could be generated at the defect area to aid the tendon–bone repair.^[^ ^42^ ^]^ Throughout immersion in PBS buffer, an obvious biodegradation tendency along with the damaged membrane structures was observed in all three membranes (Figure 3I; Figure S4B, Supporting Information). The 1000C/nG‐ES/St group had the highest mass loss over 35 wt.% after 56 days, in comparison to 500C‐ES (∼23 wt.%) and 500C/nG‐ES/St (∼20 wt.%), which could be attributed to the increased GelA content (the increased thickness of the microporous layer) and the lowest mechanical stability. Intriguingly, the surface porosity and pore size of the microporous layer were enhanced a lot by the corrosive fluid (Figure S4B, Supporting Information), which should be useful to allow the tendon fiber insertion at the late stage of repair.^[^ ^1^, ^9^ ^]^ Given the enzymatic‐degradation nature of the PCL‐GelA polymer blend used, it is anticipated that the degradation of the as‐fabricated membrane will be more rapid in vivo than in PBS buffer and meet a complementary balance with the tendon–bone healing process.^[^ ^43^, ^44^, ^45^ ^]^

### 2.6 In Vitro Biocompatibility and Biofunctions of the Tissue Mimetic Membranes

**Figure 4:**
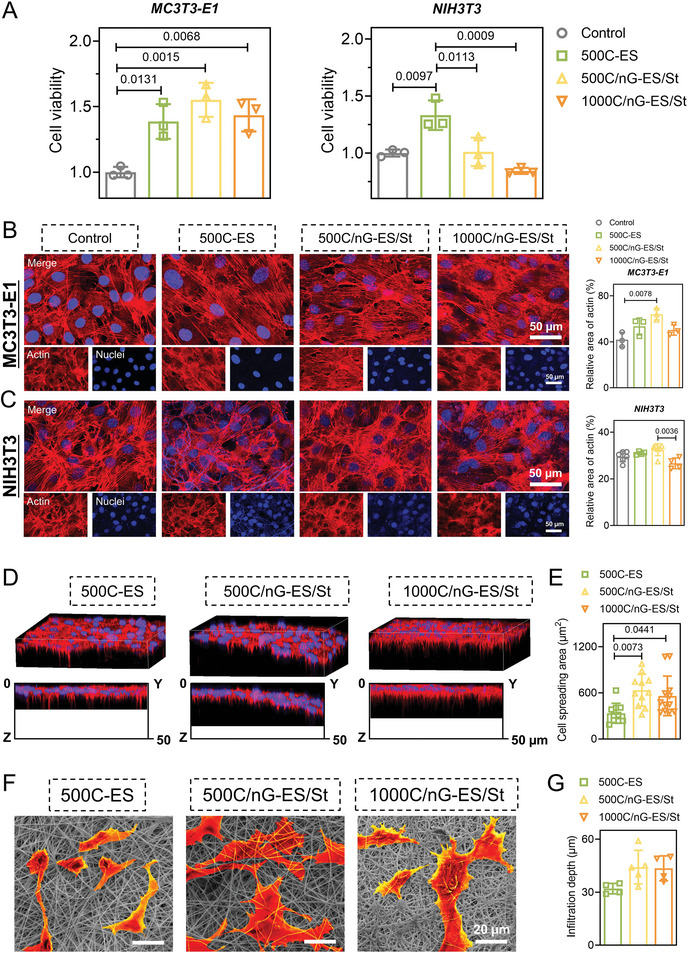
In Vitro biocompatibility of the fabricated tissue mimetic membranes – 500C‐ES, 500C/nG‐ES/St, and 1000C/nG‐ES/St. A) Viability of MC3T3‐E1 preosteoblasts or NIH3T3 fibroblasts cultured on the membranes for 3 days via MTT assay (*n* = 3). B) Cytoskeleton imaging and corresponding quantification of actin filaments of MC3T3‐E1 cells after a 3‐day incubation (*n* = 3). C) Cytoskeleton imaging and corresponding quantification of actin filaments of NIH3T3 cells on the tissue mimetic membranes after a 3‐day incubation (*n* = 4–6). D) Representative 3D and 2D cross‐sectional fluorescent images of MC3T3‐E1 cells cultured on the fibrous layers of the tissue mimetic membranes for 3 days. The membrane surfaces were set at *z* = 0. E) Corresponding quantification of cellular infiltration depth (*n* = 4–5). F) Representative SEM images of MC3T3‐E1 cells cultured on the fibrous layers of the tissue mimetic membranes for 3 days. The pseudocolor was added to the cells using ImageJ software. G) Quantification of spreading areas of the MC3T3‐E1 cells cultured on the fibrous surfaces of the three membranes for 3 days (500C‐ES: *n* = 10–12). A tissue culture plate was set as the control group. All quantitative data are presented as the mean ± SD. One‐way ANOVA with *Tukey's post hoc* test (A,B,C,E,G) was used.

Regenerative scaffolds must provide a favorable microenvironment to ensure essential cellular activities.^[^ ^46^ ^]^ To verify the biofeasibility of the as‐fabricated tissue mimetic membranes, we first evaluated the cytocompatibility by cell viability assay and cytoskeleton staining. After a 3‐day co‐culture, either MC3T3‐E1 preosteoblasts or NIH3T3 fibroblasts showed high cell viabilities as well as healthy morphologies and good cellular adherence in all three groups, indicating excellent biocompatibility (**Figure** **4**A–C).^[^ ^29^, ^47^ ^]^ The mineralized fibrous layer is expected to be a conductive platform for the homing of stem cells upon implantation. Hence, we further investigated the cell infiltration and attachment behaviors by seeding MC3T3‐E1 cells on the fibrous surface. Obvious cellular infiltration was observed after the 3‐day incubation in all three groups, where cells grew on the struvite/nGel‐KGN‐incorporated membranes (500C/nG‐ES/St: ∼44.2 µm; 1000C/nG‐ES/St: ∼43.5 µm) displayed a larger infiltration depth compared to the pure polymer membrane (500C‐ES: ∼32 µm) (Figure 4D,E). Moreover, remarkably enlarged cell spreading area was also found on 500C/nG‐ES/St and 1000C/nG‐ES/St membranes in comparison with 500C‐ES membrane, demonstrating the promotive effect of the bioactives on cell–material integration (Figure 4F,G). The scratch wound assay showed that the migration of NIH3T3 cells was significantly enhanced in the 500C/nG‐ES/St group as compared to another group after being cultured for 24 h, which could potentially aid in the progression of tendon–bone healing (Figure S5A,B, Supporting Information).^[^ ^48^ ^]^ In our design, the microporous layer was put near the tendon during in vivo repair to exclude the invasive fibrovascular scar tissue at an early stage.^[^ ^1^ ^]^ After a 5‐day culture, the NIH3T3 fibroblasts grew well on all three microporous surfaces, while no cell was found on the opposite fibrous surfaces, demonstrating the robust barrier effect. More encouragingly, the attached fibroblasts displayed flattened and spread morphologies with a cellular layer formation, especially in the 500C/nG‐ES/St group (Figure S5C, Supporting Information). Given the normal tendon is populated by elongated fibroblasts interspersed within aligned collagen fibrils, the unmineralized microporous layer shows great potential to guide tendon‐to‐membrane ingrowth at the late stage of implantation.^[^ ^49^ ^]^

### 2.7 In Vitro Osteo‐ and Chondro‐Promotive Effect of the Tissue Mimetic Membranes

**Figure 5:**
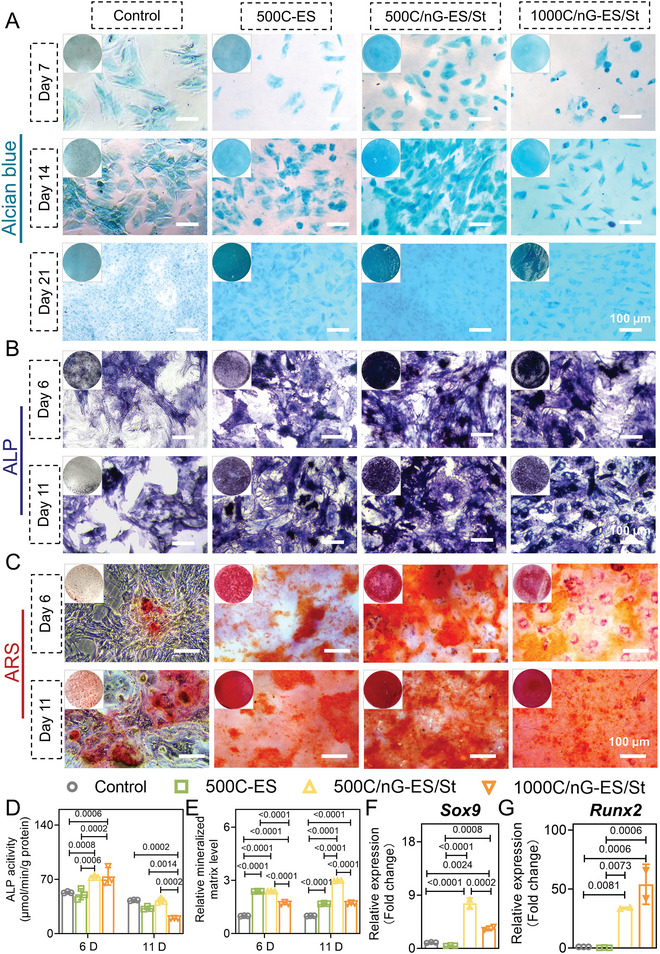
Bi‐lineage bioinducible effect of the fabricated tissue mimetic membranes. A) Alcian blue staining of the samples on days 7, 14, and 21 post‐incubation. B) Alkaline phosphatase (ALP) staining of the samples on days 6 and 11 post‐incubation. C) Alizarin Red S (ARS) staining of the samples on days 6 and 11 post‐incubation. D) ALP activity of rBMSCs on the membranes on days 6 and 11 post‐incubation (*n* = 3). E) Quantitative analysis of calcium deposition of the samples on days 6 and 11 post‐incubation (*n* = 3). F) Relative expression of *Sox9* in rBMSCs after a 7‐day incubation (*n* = 2–3). G) Relative expression of *Runx2* in rBMSCs after a 7‐day incubation (*n* = 2–3). A tissue culture plate was set as the control group. All quantitative data are presented as the mean ± SD. Two‐way ANOVA with *Sidak's post hoc* test (D,E) was used. One‐way ANOVA with *Tukey's post hoc* test (F,G) was used.

Herein, rat bone mesenchymal stem cells (rBMSCs) were utilized to investigate the osteo‐/chondrogenic bi‐lineage promotive effect of the materials. Alcian blue selectively binds with the cartilaginous extracellular matrix and is commonly implemented for chondrocyte confirmation.^[^ ^50^ ^]^ The alcian blue‐positive cells were found on all three types of membranes on days 7, 14, and 21 post‐incubation, and the staining intensity increased with the incubation time, indicating the progression of chondrogenesis. Notably, the 500C/nG‐ES/St group exhibited the densest blue stain and the highest positive‐stained cell density during the observation (**Figure** **5**A). As for the osteogenesis behaviors of rBMSCs, 500C/nG‐ES/St and 1000C/nG‐ES/St groups demonstrated significantly enhanced alkaline phosphatase (ALP) activity, the early‐stage osteogenic marker, after the 6‐day incubation, evidenced by the increased stain area and the higher ALP activity values compared to control or 500C‐ES group. The overall ALP activity in all groups slightly decreased from day 6 to day 11, whereas it is noteworthy that the 500C/nG‐ES/St group consistently exhibited remarkably enhanced ALP activity and the most intense purple stain at both time points (Figure 5B,D).^[^ ^51^ ^]^ Further, Alizarin Red S (ARS) staining was applied to visualize the ECM mineralization, a late‐stage osteogenic biomarker. In line with the findings regarding ALP activity, the 500C/nG‐ES/St group displayed the deepest red stain along with the highest mineralized level across the groups, which was particularly prominent on day 11 (Figure 5C,E), demonstrating the substantially optimized osteogenesis, which is likely attributed to the presence of osteo‐promotive struvite (specifically, the degradation products, Mg^2+^ and phosphate ions [PO_4_^3−^]).^[^ ^18^, ^51^ ^]^ The expression of *Sox9* guides stem cell differentiation toward chondrogenic lineage, while Runt‐related transcription factor 2 (*Runx2*) plays a critical role in chondrocyte maturation as well as the endochondral bone formation.^[^ ^52^, ^53^ ^]^ After a 7‐day induction, the expressions of *Sox9* and *Runx2* were remarkably upregulated in 500C/nG‐ES/St and 1000C/nG‐ES/St groups compared to the control or 500C‐ES group (Figure 5F,G). However, further increasing the dose of nGel‐KGN might induce an adverse effect, as evidenced by the suppressed expression of *Sox9* and the decreased bi‐lineage staining in 1000C/nG‐ES/St as compared to 500C/nG‐ES/St. This could be attributed to the negative impact of an excessive amount of nGel‐KGN particles on stem cell proliferation and aggregation, consequently impairing differentiation.^[^ ^16^ ^]^ In summary, the released nGel‐KGN and struvite could contribute to an inductive microenvironment to promote the bi‐lineage differentiation of stem cells, while more in vivo evidence is required to investigate the specific dosage effect of nGel‐KGN.

### 2.8 Evaluation of the Repair of Tendon–Bone Interface in Rotator Cuff Tear (RCT) Rats

**Figure 6:**
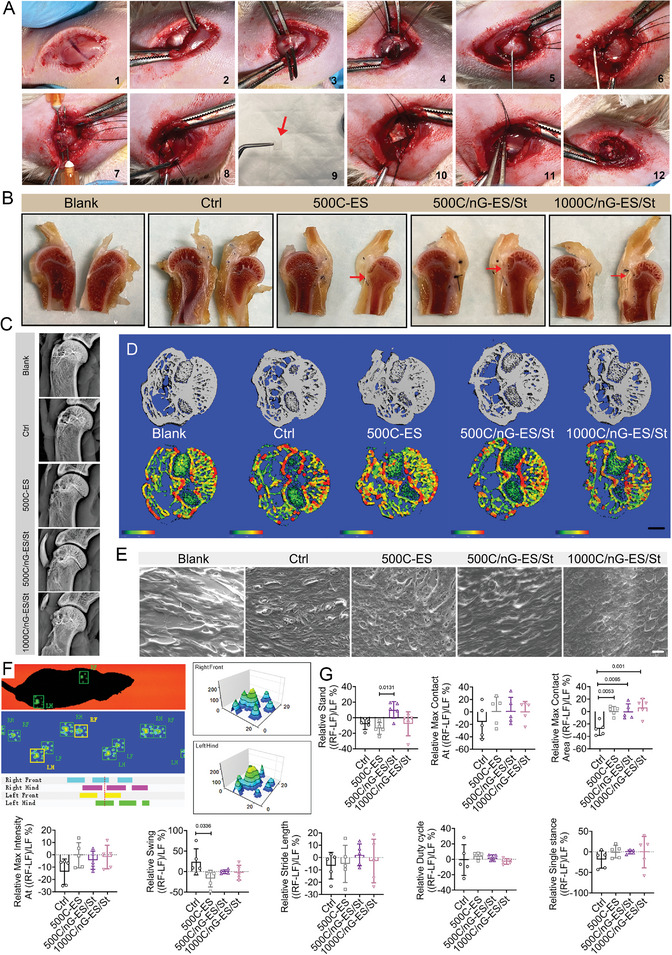
Application and evaluation of the tissue mimetic membranes in the repair of tendon–bone interface in RCT rats. A) Illustration of the RCT surgical procedure and membrane implantation. B) Representative macroscopic images of the anatomical plane in the membrane implantation at week 8 post‐surgery. The red arrow indicates the membrane‐implanted site. C) Representative X‐ray images of the humerus in rats at week 8 post‐surgery. D) Reconstructed 3D micro‐CT images of cross‐section in proximal humerus at week 8 post‐surgery. Scale bar: 1 mm. E) Representative SEM images of ingrowth cells and well‐aligned collagen formation on the implanted membranes at week 8 post‐surgery. Scale bar: 10 µm. F) Automatic footprints and gaits captured by the catwalk gait analysis system. G) Representative catwalk gait analysis across groups at week 8 post‐surgery. *n* = 5 rats per group. LF: Left forelimb; RF: right forelimb; LH: left hindlimb; RH: right hindlimb. Max Contact Area: Maximal contact area; Max Contact AT: maximal contact AT; Max Intensity At: maximal intensity AT. All quantitative data are presented as mean ± SD. One‐way ANOVA with *Tukey's post hoc* test (G) was used.

We further investigated the regenerative efficacy of the tissue mimetic membranes for the repair of tendon–bone interface using an RCT model in rats. The membrane was implanted at the supraspinatus tendon–bone interface with the microporous layer facing the tendon (**Figure** **6**A). At week 8 post‐implantation, macroscopic images revealed a successful integration of the membrane with surrounding tissues (Figure 6B). In 1000C/nG‐ES/St group, the presence of residual membrane correlated with chronic bone erosion and remodeling, suggesting that the 500 µm‐casting microporous layer (∼46 µm) is more optimal for the tendon ingrowth, while the increase of the thickness to ∼113 µm (1000 µm‐casting microporous layer) retarded this process (Figure S1J, Table S1, Supporting Information). Histological analysis of major visceral organs at week 8 post‐implantation showed no morphological alterations in different groups, verifying the favorable in vivo biocompatibility (Figure S6, Supporting Information). The X‐ray and micro‐CT imaging of the humerus indicated enhanced bone regeneration along with superior tissue–material integration in the 500C/nG‐ES/St group as compared to the other groups, implying the high efficiency in stimulating the bony ingrowth toward the tendon–bone interface (Figure 6C,D). Additionally, SEM analysis showed cellular proliferation within the membrane layers, and cells displayed adaptability across all membrane‐treated groups, especially in the 500C/nG‐ES/St group (Figure 6E). Despite similarities in certain Cat‐walk apparatus parameters across groups, the significant differences in relative Stand, Max Contact Area, and Swing revealed notably improved functional behaviors in the 500C/nG‐ES/St group compared to the control group (Figure 6F,G). Therefore, the 500C/nG‐ES/St membrane provided the most effective augmentation for the restoration of the normal function of the rotator cuff.

**Figure 7:**
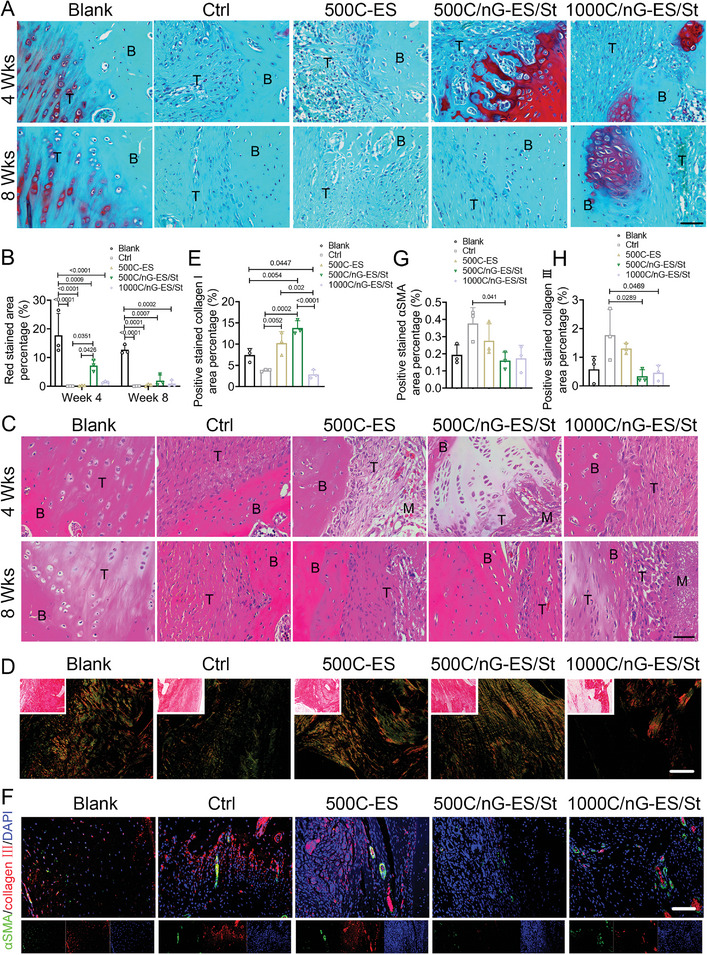
The in situ regeneration of tendon–bone interface with the tissue mimetic membrane in RCT rats. A) Representative Safranin O/Fast green staining and B) quantifications of positive stained red area at weeks 4 and 8 post‐surgery. *n* = 3 rats per group. B: bone; T: tendon. Scale bar: 50 µm. C) Representative hematoxylin and eosin (H&E) staining of tendon–bone interface at weeks 4 and 8 post‐surgery. B: bone; T: tendon; M: membrane. Scale bar: 50 µm. D) Representative Picro‐Sirius Red staining and E) quantification of collagen I at the tendon–bone interface at week 8 post‐surgery. Scale bar: 100 µm. F) Representative images and G,H) quantifications of immunofluorescence co‐staining of alpha‐smooth muscle actin (𝛼SMA) and collagen III at tendon‐bone interface at week 8 post‐surgery. *n* = 3 rats per group. Scale bar: 100 µm. All quantitative data are presented as mean ± SD. Two‐way ANOVA with *Sidak's post hoc* test (B) was used. One‐way ANOVA with *Tukey's post hoc* test (E,G,H) was used.

**Figure 8:**
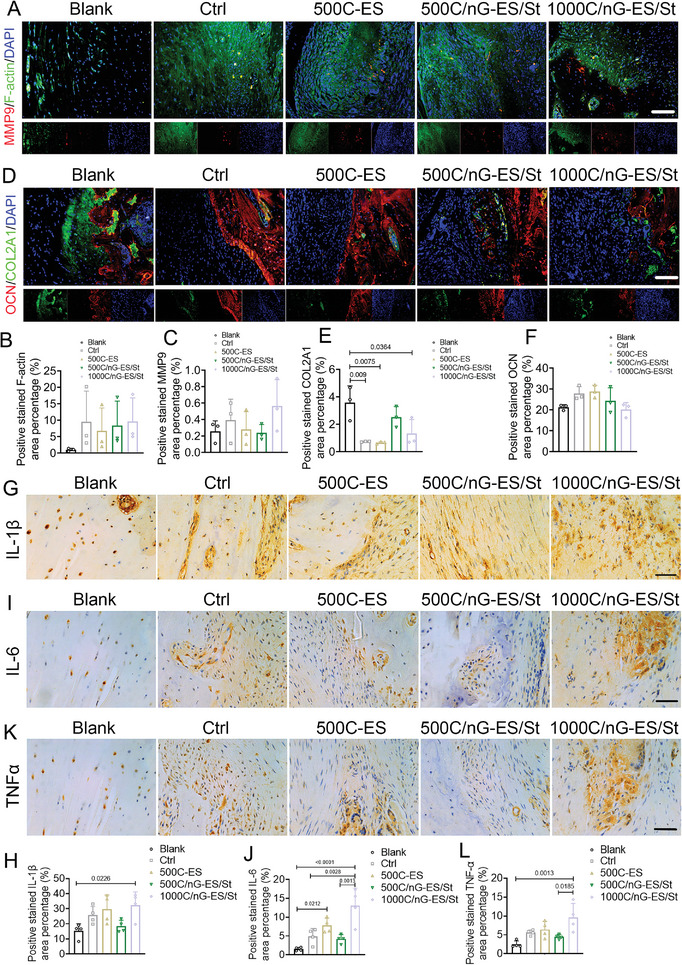
Matrix synthesis and inflammatory responses at tendon–bone interface. A) Representative images and B,C) quantifications of immunofluorescence co‐staining of F‐actin and MMP9 at tendon–bone interface at week 8 post‐surgery. *n* = 3 rats per group. Scale bar: 100 µm. D) Representative images of immunofluorescence co‐staining of OCN and COL2A1 and E,F) quantification of (E) COL2A1 and (F) OCN staining at tendon‐bone interface at week 8 post‐surgery. *n* = 3 rats per group. Scale bar: 100 µm. G,I,K) Representative images and H,J,L) quantifications of immunohistochemical staining of IL‐1β, IL‐6, and TNF‐α at tendon‐bone interface at week 8 post‐surgery. *n* = 4 rats per group. Scale bar: 50 µm. All quantitative data are presented as mean ± SD. One‐way ANOVA with *Tukey's post hoc* test (B,C,E,F,H,J,L) was used.

To deepen our understanding of membrane‐mediated tendon–bone healing, the corresponding histological and immunofluorescence analysis of the key biomarkers were performed (**Figure** **7**). Safranin O/Fast green staining was implemented to assess the regeneration of the fibrocartilaginous interface, which largely determines the success of tendon–bone repair.^[^ ^15^ ^]^ 500C/nG‐ES/St and 1000C/nG‐ES/St groups exhibited the formation of the fibrocartilaginous matrix (positively stained with Safranin O/Fast green) at the tendon–bone interface, which was particularly notable at week 4 post‐RCT surgery (Figure 7A,B). Rotator cuff healing and membrane degradation were evaluated at weeks 4 and 8 through hematoxylin and eosin (H&E) staining (Figure 7C; Figure S7, Supporting Information). Notable decomposition and partial dissolution of the membranes were observed in all implanted groups, implying favorable degradation in vivo (Figure S7, Supporting Information). All membrane‐treated groups showed hypercellular infiltration (Figure 7C). However, bone loss appeared at the tendon–bone interface in 1000C/nG‐ES/St at week 8, which may be attributed to the inflammatory clearance related to the prolonged phagocytosis.^[^ ^54^ ^]^ During the early healing, extracellular matrix (ECM) deposition by fibroblasts is rich in collagen III, which is more related to scar formation and then replaced by collagen I at the later stage.^[^ ^55^ ^]^ Picro‐Sirius red staining was applied to assess the collagen organization and maturity at the defect area, where collagen I displayed yellow/orange birefringence and collagen III exhibited green birefringence under polarized light.^[^ ^56^ ^]^ After the 8‐week implantation, the tendon repaired with the 500C/nG‐ES/St membrane exhibited denser and more organized collagen I deposition as compared to the other RCT groups (Figure 7D,E). Immunofluorescence co‐staining revealed the significant reductions of collagen III and alpha‐smooth muscle actin (𝛼SMA, a representative myofibroblast marker) in 500C/nG‐ES/St and 1000C/nG‐ES/St groups, compared to control and 500C‐ES groups at week 8 post‐surgery (Figure 7F–H). Besides, although the expression of F‐actin, a cytoarchitectural protein, was comparable among the RCT groups at week 8, the presence of the tissue mimetic membrane could greatly optimize the cellular ingrowth to the defect area (**Figure** **8**A,B; Figure S7, Supporting Information). Upregulated expression of metallopeptidase 9 (MMP9) was observed in the 1000C/nG‐ES/St group, which is a class of enzyme belonging to the zinc‐metalloproteinases family and plays a crucial role in the degradation of extracellular matrix during tissue remodeling (Figure 8A,C).^[^ ^57^ ^]^ Osteocalcin (OCN) is a key marker for mature osteoblasts, which contributes to the maturation of hydroxyapatite crystals.^[^ ^58^ ^]^ However, the expression of OCN was comparable among all groups. Notably, rather more newly formed collagen II was found in the 500C/nG‐ES/St group than in other RCT groups, suggesting the substantially enhanced biosynthesis of cartilage matrix at the tendon–bone insertion site (Figure 8D–F).^[^ ^42^ ^]^ Inflammation aids debris clearance and signals tissue regeneration. Immune cells and their functional phenotypes may be the key to tendon–bone healing.^[^ ^59^, ^60^ ^]^ We further analyzed the expression of the representative inflammatory markers (i.e., IL‐1β, IL‐6, and TNF‐α) at week 8 post‐surgery at the repaired interface. An increased secretion of inflammatory cytokines was observed in the RCT groups compared to the blank group (i.e., normal tissue), which was particularly significant in the 1000C/nG‐ES/St group (Figure 8G–L). Given the excess inflammatory response can act as a risk factor, predisposing to a failed healing response,^[^ ^61^, ^62^ ^]^ the elevated inflammatory response in the 1000C/nG‐ES/St group may exert a negative impact to a certain degree during the repair process, as revealed by the H&E findings (Figure 7C).

To summarize, at week 8 post‐surgery, the RCT group without membrane augmentation (i.e., the control group) exhibited obvious fibrovascular tissue formation, inflammatory cell infiltration, and irregular collagen arrangement at the lesion. The application of the biphasic tissue mimetic membranes could remarkably suppress scar tissue formation and optimize the regeneration of fibrocartilaginous tissue at the tendon–bone interface. Notably, the 500C/nGel‐ES/St group exhibited enhanced cellular recruitment and bi‐lineage differentiation, accelerated bony ingrowth, as well as well‐organized collagen fiber formation, surpassing the outcomes observed in other RCT groups. In line with the In Vitro findings, a negative dose‐response of nGel‐KGN particles was also observed in vivo, indicating the existence of a threshold dosage. In addition, a more than 2‐fold increase in the thickness of the microporous layer significantly deteriorated the interface tissue regeneration, likely due to the restrained tendon‐to‐membrane integration and aggravated inflammatory responses.

## 3 Discussions

In this study, we developed a tissue‐mimetic membrane for implantation at the injury site to enhance the functional reconstruction of the transitional enthesis and restore normal shoulder functions following rotator cuff tears. To stimulate the interface fibrocartilaginous tissue regeneration and enhance tendon‐to‐bone integration, a biomimicry heterogeneous topography, a sharp mineral gradient, and bi‐lineage bioinductive chemical cues were incorporated within a single membrane. The In Vitro and in vivo findings validated the effectiveness of the as‐fabricated tissue mimetic membrane, in particular, the 500C/nG‐ES/St membrane, for the healing augmentation of tendon–bone interface as well as the chronological and spatial modulations of the healing events. This study offers a promising approach to developing tendon–bone healing strategies.

Tendon–bone interface is a typical complex region organized into distinct zones – tendon, unmineralized and mineralized fibrocartilage, and bone. This gradual transition allows for strong bonding and a proper force dissipation between the soft and hard tissues.^[^ ^63^ ^]^ Current tissue‐engineering strategies strive to recapitulate the functional gradient interface during rotator cuff repair. Given the structure‐function relationship, the biomimetic anisotropic structure of native tissues is advantageous.^[^ ^1^ ^]^ Biological‐based scaffolds (e.g., the decellularized extracellular matrix (ECM) scaffolds), which exhibit inherent biomimicking structural characteristics, are extensively investigated for RCT repair. Several products have been clinically available like the human dermis‐derived GraftJacket regenerative tissue matrix (LifeCell, Wright Medical Technology, USA) and the porcine small intestine submucosa‐derived Restore orthobiologic augmentation patch (DePuy Orthopaedics, USA).^[^ ^64^ ^]^ However, the residual immunogenic components are prone to induce immune response, usually followed by inflammation/rejection and a failed repair. These implants are also associated with the limited availability of suitable donor tissues. To address these limitations, we constructed a biphasic membrane that simulated the transitional unmineralized (microporous layer) and mineralized (fibrous layer) regions between tendon and bone, using biocompatible polymers through controlled membrane fabrication strategies. The as‐fabricated membrane offered a tissue mimetic supportive platform for endogenous MSCs to aggregate, proliferate, and differentiate with the coordination of specific chemical cues, as well as provided a topographical cue for tendon‐to‐membrane and bone‐to‐membrane ingrowth, ultimately promoting tendon‐to‐bone re‐attachment (Scheme 1). Besides, the restricted porosity of the upper microporous layer suppressed the invasion of fibrovascular scar tissues at the early stage, thereby also contributing to the enhanced tendon–bone healing.

The failure of spontaneous fibrocartilage regeneration after tendon–bone injury is closely related to the limited number of stem cells at the lesion site and the restrained ability of them to differentiate toward the lineages of the enthesis concurrently.^[^ ^45^, ^65^ ^]^ Therefore, in addition to the biophysical cue, it would be more beneficial to combine biochemical/biological stimuli to trigger the *in‐situ* stem cell differentiation into desired lineages. The incorporation of exogenous growth factors or small‐molecule drugs endows the scaffold with a specific therapeutic effect. However, a significant challenge is to find an appropriate vehicle that can ensure sustained delivery of the therapeutic agents at a suitable concentration and for a sufficient duration.^[^ ^63^ ^]^ Previously, we have successfully fabricated poly (N‐isopropylmetharylamide) (PNIPMAM) nGel/siRNA conjugates through a “breath‐in” method, demonstrating a robust curative efficacy to mitigate osteoarthritis (OA) progression in mice.^[^ ^24^ ^]^ Inspired by this, we synthesized an amino‐functionalized nanogel as the carrier of the insoluble KGN and successfully fabricated the cationic nGel‐KGN conjugates as the chondroinductive cues. A sustained release of nGel‐KGN conjugates was achieved, contributing to the significantly enhanced chondrogenesis of endogenous MSCs. The upregulated expression of cartilaginous marker (COL2A1), the formation of denser and more organized collagen fibers (collagen I), and ultimately the accelerated regeneration of fibrocartilaginous matrix was observed during the implantation. Given the high re‐rupture rate is closely related to the restrained regenerative capacity of the transitional fibrocartilaginous tissues after tendon–bone injury, the boosted chondrogenesis by nGel‐KGN would benefit the tendon–bone repair.^[^ ^15^ ^]^ The biomechanical strength of the repaired tendon–bone interface is positively correlated with the osseous ingrowth.^[^ ^66^ ^]^ Struvite was prone to degrade in the aqueous environment to release Mg^2+^ and PO_4_^3−^, which were found to actively promote the osteogenic differentiation of MSCs and bone matrix mineralization.^[^ ^67^, ^68^ ^]^ The prolonged release of struvite from the core‐shell nanofibrous matrix led to enhanced bone formation at the defective interface and ultimately the optimized restoration of functional motion of the rotator cuff.

Of note, there are some limitations in the present study. First, the follow‐up period is relatively short (i.e., 8 weeks). The maturation and organization of the interface tissue continue to progress from 8 weeks onward, often extending beyond 12 weeks.^[^ ^45^ ^]^ We observed the formation of the fibrocartilaginous matrix in the 500C/nG‐ES/St and 1000C/nG‐ES/St groups, but a structurally integrated transition zone was not fully achieved after an 8‐week implantation. Hence, an extended observation period may be beneficial to gain more insights into the repair capacity of the tissue mimetic membranes. Second, we utilized the In Vitro results and dissected the membrane‐mediated regeneration to unveil the in vivo release process, whereas it would be more advantageous to directly profile the *in‐situ* release tendency during implantation. Third, it will be meaningful to further optimize the membrane thickness as well as the loading content of nGel‐KGN and struvite for better rotator cuff repair, by considering the bioinducible effect, tissue integration, and the regenerative capacity of fibrocartilaginous tissues. Finally, the microporous layer induced by particle agglomeration displayed low mechanical stability, which would impair the mechanical performance of the membrane when its proportion significantly increased. Though the implantation site (between tendon and bone) in this study was not subjected to load, further refining the porous structure to gain higher mechanical strength will lead to more application forms (like a patch bridging the massive defect).^[^ ^9^ ^]^

Despite these limitations, this study demonstrates that the bi‐lineage bioinducible tissue membrane (i.e., 500C/nG‐ES/St) is a promising clinical solution for the functional repair of the rotator cuff. This membrane displays a sustained therapeutic effect over the implantation period and has addressed the major restraints of existing products, including the excess inflammatory responses in allogenic GraftJacket regenerative tissue matrix and the lack of bioactivity in synthetic X‐repair membranes.^[^ ^9^, ^64^ ^]^ Beyond the application in RCT repair, such asymmetric membranes that center on recapitulating the anisotropic aspects of native tissues and promoting multi‐tissue regeneration hold great potential for the functional reconstruction of other complex musculoskeletal regions, like the osteochondral interface in articulating joints.^[^ ^69^, ^70^ ^]^ Additionally, the developed biphasic membrane displays superior barrier performance (i.e., excluding the tissue interference) and osteogenic inductive capacity, making it a promising material for guided bone regeneration (GBR) applications in dental clinics.^[^ ^71^ ^]^

## 4 Conclusion

To optimize the outcome following rotator cuff surgery, a tissue mimetic membrane has been successfully developed for healing augmentation of the fibrocartilaginous tendon–bone interface. The as‐fabricated membrane was constituted by an nGel‐KGN‐laden microporous layer and a struvite‐mineralized core‐shell nanofibrous layer, that simulated the interfacial heterogeneity, as well as generated bi‐lineage bioinducible effect. In a rat rotator cuff tear model, the tissue mimetic membrane prominently promoted regenerative outcomes at tendon–bone interface. The 500C/nG‐ES/St membrane with 4% St. in the fibrous layer and 5% nGel‐KGN in ∼45.7 µm‐thick microporous layer demonstrated the best regenerative performance. Building on the promising In Vitro and in vivo results, it is anticipated that the presented tissue mimetic membrane can potentially be an effective clinical solution to address the interface tissue deficiency at the rotator cuff and even other transitional regions in sports medicine.

## 5 Experimental Section

### Synthesis of Positive‐Charged Ketogenic‐Conjugated Nanogel (nGel‐KGN) Particles

The synthesis route of the nGel‐KGN particles is shown in Figure 1A. First, the fluorescent amino‐functionalized poly (N‐isopropylmetharylamide) (PNIPMAM) nGel particles were synthesized via free‐radical precipitation polymerization followed by seeded precipitation polymerization, as previously reported.^[^ ^24^ ^]^ In a typical reaction, 137.2 mM N‐isopropylmetharylamide (NIPMAM, Sigma, cat# 423548), 2.8 mM N,N’‐methylenebis(acrylamide) (BIS, Sigma, cat# 146072), and 8 mM sodium dodecyl sulfate (SDS, TCI, cat# I0352) were first dissolved in 100 mL deionized water (D. I. H_2_O) to obtain a homogenous solution. The solution was then heated to 70 °C under a N_2_ atmosphere in a reaction flask. Subsequently, 0.1 mM fluorescein O‐acrylate (FL., Sigma, cat# 568856) was dissolved to obtain a solution in light green color. After the temperature was stable at 70 °C for a further 30 min under an N_2_ atmosphere, 1 mL of 0.8 M ammonium persulfate (APS, TCI, cat# A2098) solution was injected to initiate the polymerization reaction. After 4 h, the polymerization was terminated, and the product was purified using a 0.22 µm syringe filter. Then, the as‐synthesized nanogel core particles were utilized as the seeds for the growth of an amino‐functionalized polymer shell. Typically, 48.75 mM NIPMAM, 1 mM BIS, and 0.25 mM *N*‐(3‐aminopropyl methacrylate hydrochloride (AMPA, Alfa Aesar, cat# Y11329) were dissolved in 39.5 mL D. I. H_2_O to obtain a monomer solution. 0.0577 g SDS was dissolved in 10 mL nanogel core solution in a reaction flask. Then, the prepared monomer solution was transferred to the flask under vigorous stirring and heated to 70 °C under an N_2_ atmosphere. After the temperature was stable for 30 min, 1.5 mL of 0.05 Mm APS solution was added to initiate the polymerization reaction. After 4 h, the reaction was terminated by cooling down the system. The product was first purified via a 0.22 µm syringe filter and then dialyzed (MWCO: 12–14 kDa) in D. I. water for 3–5 days to remove any unreacted molecules and short‐chain polymers. After dialysis, the solution was further filtered by a 0.22 µm syringe filter and then lyophilized to obtain the dry fluorescent amino‐functionalized nGel particles. Then, the KGN molecule was conjugated to the surface of nGel particles via an EDC/NHS coupling reaction. Briefly, 88.3 mg nGel particles were dissolved in 35 mL N, N‐dimethyl formamide (DMF, Merck, cat# 200‐679‐5) to prepare a homogeneous suspension. 1.4 mg KGN, 0.84 mg 1‐(3‐dimethylaminopropyl)‐3‐ethylcarbodiimide hydrochloride (EDC∙HCl, TCI, cat# D1601), and 0.51 mg N‐Hydroxysuccinimide (NHS, Sigma cat# 130672) were dissolved in 3 mL DMF and shaken for 2 h at room temperature. Then, this solution was added to the nGel suspension and reacted at room temperature on a shaker overnight. After the reaction, the product was dialyzed (MWCO: 12–14 kDa) in D. I. H_2_O for 3–5 days for purification. The purified solution was then lyophilized to obtain the dry nGel‐KGN particles. Subsequently, the nanogel‐KGN particles were coated with poly (ethyleneimine) (PEI) polymer by simple mixture and sonication.^[^ ^16^ ^]^ Typically, 4 mg mL^−1^ nGel‐KGN particles and 1 mg mL^−1^ PEI (branched, Mw. ∼25,000, Sigma, cat# 408727) were mixed in D. I. H_2_O and sonicated for 5 min. Then, the mixture was dialyzed (MWCO: 50 kDa) in D. I. H_2_O for 5 days to remove any free PEI polymers. After purification, the product was lyophilized to obtain the positive‐charged nGel‐KGN particles, which were stored at 4 °C for further use.

### Characterization of nGel‐KGN Nanoparticles

The nGel‐KGN particle morphology was examined using transmission electron microscopy (TEM, Tecnai G2 Spirit Bio, FEI, USA). The lyophilized nGel‐KGN particles were first suspended in absolute ethanol. The dispersion was subsequently dropped onto the carbon‐coated copper grid and dried in the air. The TEM observation was performed at the accelerated voltage of 120 kV. Further, the size (diameter) distribution of the dry nGel‐KGN particles was analyzed based on TEM images using ImageJ software (version 1.52k).

The hydrodynamic size distribution of the nGel‐KGN particles was determined via a dynamic light scattering analyzer (ZetaSizer Nano ZS90, Malvern Panalytical, Malvern, UK) at 25 and 37 °C to examine the phase stability in the physiological environment. The lyophilized nanogel particles were dispersed in 2.5 mM HEPES buffer and ultrasonicated for 20 min to prepare a homogeneous suspension at the concentration of 5 mg mL^−1^. The suspension was put into the chamber and allowed to equilibrate for 5 min before each measurement. The measurements were performed at a fixed scattering angle of 90°. Particle zeta potential of nGel‐KGN with or without PEI was measured using the same instrument at 37 °C. Nanoparticle tracking analysis (NTA) measurements of the nGel‐KGN particles in 2.5 mM HEPES buffer at 1.25 mg mL^−1^ were performed with a NanoSight LM10 (NanoSight, Amesbury, UK) at 25 and 37 °C. The video frame of nGel‐KGN particles was extracted via the ImageJ software (version 1.52k).

The UV–vis spectrum of KGN, nGel, and nGel‐KGN was measured using a UV–vis spectrometer (UV‐3600 Plus, Shimadzu, Japan). The KGN, nGel, and nGel‐KGN were dispersed in DMSO and ultrasonicated for 10 min to prepare a homogeneous and transparent suspension. The concentrations of nGel and nGel‐KGN suspensions were 5 mg mL^−1^. The UV–vis spectrum was recorded from 450 to 220 nm at a scanning rate of 0.5 nm s^−1^. The fluorescent spectrum of the nGel‐KGN particles was measured using a fluorescent spectrometer (F‐7000, Hitachi, Japan). The nGel‐KGN particles at different concentrations (0.1, 0.25, 0.5, 1, 2.5, and 3.75 mg mL^−1^) were dispersed in PBS buffer and ultrasonicated for 10 min to prepare a homogeneous suspension. The fluorescent emission spectrum was recorded from 400 to 700 nm at the excitation wavelength of 450 nm.

The chemical structures of KGN, nGel, and nGel‐KGN were examined by ^1^H NMR analysis. The ^1^H NMR samples were prepared by dissolving in deuterated DMSO and then analyzed using an AVANCE III 400 MHz NMR Spectrometer (Bruker, Germany).

### Determination of the Binding Degree of KGN Molecules onto the nGel Surface

48.5 mg nGel particles were dissolved in 19.2 mL N, N‐dimethyl formamide (DMF, Merck, cat# 200‐679‐5) to prepare a homogeneous suspension. 0.769 mg KGN, 0.46 mg 1‐(3‐dimethylaminopropyl)‐3‐ethylcarbodiimide hydrochloride (EDC∙HCl, TCI, cat# D1601), and 0.28 mg N‐Hydroxysuccinimide (NHS, Sigma cat# 130672) were dissolved in 1.6 mL DMF and shaken for 2 h at room temperature. Then, this solution was added to the nGel suspension and reacted at room temperature on a shaker overnight. After the reaction, the unreacted KGN was precipitated from DMF upon the addition of a large amount of D. I. H_2_O. Then, the precipitate (unreacted KGN) was collected by centrifugation at 10000 G for 30 min and dried in the air. After drying, the unreacted KGN was dissolved into 24 mL DMSO, and the concentration was determined using UV–vis spectra. First, the standard solutions of KGN at the concentrations of 0, 0.001, 0.0025, 0.005, 0.0075, and 0.01 mg mL^−1^ (in DMSO) were prepared and the absorbance of the standard solution at 277 nm was measured respectively using UV–vis spectrometer. The standard curve of absorbance at 277 nm (Abs.) vs concentration (Conc.) of KGN was obtained: Abs. = 49.28788 Conc. (R^2^ = 0.99974) Subsequently, the UV spectra of the unreacted KGN solution were recorded from 450 to 240 nm, and the absorbance at 277 nm was obtained from the spectra (Abs_277_ = 0.29643). Then, the amount of unreacted KGN was calculated based on the standard curve, and the binding degree was calculated according to Equation 1.

(1) $$\begin{array}{ccc} & & \mathrm{Binding}\;\mathrm{degree}\;\left(\mathrm{mol}\;g^{-1}\right)=\frac{n_{\mathrm{reacted}\;\mathrm{KGN}}}{m_{\mathrm{nGel}}}\\ & & \;=\frac{(m_{\mathrm{added}\;\mathrm{KGN}}\;-\;m_{\mathrm{unreacted}\;\mathrm{KGN}})/M_{w_{\mathrm{KGN}}}}{m_{\mathrm{nGel}}} \end{array}$$

### Synthesis of Struvite (St.) Nanowires

The St. nanowires were synthesized by a controlled crystallization method as previously reported.^[^ ^18^ ^]^ Briefly, solution A containing 1 mg mL^−1^ magnesium chloride hexahydrate (MgCl_2_·6H_2_O, Sigma, cat# M0250) and solution B containing 2.3 mg mL^−1^ ammonium dihydrogen phosphate (NH_4_H_2_PO_4_, Sigma, cat# 204005) and 2.1 mg mL^−1^ ammonium chloride (NH_4_Cl, Sigma, cat# 21330) were prepared. Then, 10 mL solution B was added to 40 mL solution A under continuous stirring to obtain a homogeneous solution. After that, 1.75 g sodium chloride (NaCl, Sigma, cat# S9888) was dissolved into the as‐prepared solution. Then, 10 M sodium hydroxide (NaOH) was used to adjust the pH value of the solution to ∼11.0. White precipitation gradually appeared as the pH increase and the suspension was stirred for 24 h at room temperature. Then, the product was purified using absolute ethanol (VWR, cat# EM8.18760.9180), and subsequently underwent vacuum dried at room temperature for 3 days. The obtained struvite nanowires were stored in a drying cabinet for further use.

### Characterizations of Struvite Nanowires

Crystal phase analysis of the obtained struvite was performed via an X‐ray diffractometer (XRD, SmartLab, Rigaku, Japan) at the scanning rate of 10° min^−1^, within the range from 10° to 65°. For morphological analysis, the struvite nanowires were homogeneously dispersed in absolute ethanol first, and the suspension was then dropped onto a silicon wafer or a copper grid. After drying, the surface morphology of the sample (on a silicon wafer) was determined using scanning electron microscopy (SEM, Quanta‐400, FEI, USA) at an accelerating voltage of 10 kV, while the internal structure of the sample (on copper grid) was examined by transmission electron microscopy (TEM, Tecnai G2 Spirit Bio, FEI, USA) at 120 kV. The spatial distribution of the elements in struvite nanowires was examined via scanning transmission electron microscopy (STEM, Tecnai F20 ST, FEI, USA) followed by energy X‐ray dispersive spectroscopy (EDS) at 200 kV.

### Fabrication of the Dual‐Layer Tissue Mimetic Membrane

The tissue mimetic membrane was fabricated using a non‐solvent induced phase separation (NIPS) strategy followed by co‐axial electrospinning (ES). First, the microporous layer was prepared using a polymer/nGel‐KGN mixed solution. The 15 w/v% polymer blend solution was prepared by dissolving polycaprolactone (PCL, 80 kDa, Sigma, cat# 440744) and gelatin A (GelA, ∼175 Bloom, Sigma, cat# G2625) at a weight ratio of 1:1 into a mixed solvent consisting of 1,1,1,3,3,3‐hexafluoro‐2‐propanal (HFIP, TCI, cat# H0424) and DMF at a 20:1 volume ratio, where nGel‐KGN particles were homogeneously dispersed. The prepared solution was then cast onto silicon paper with a casting blade, where the wet film thickness was set to 500 or 1000 µm. NIPS occurred during evaporation and eventually formed the microporous layer with the particle loading concentration of 0 wt.% and 5 wt.%. The obtained microporous layers were designated as 500 µm‐casting, 500 µm‐casting/5% nGel‐KGN, and 1000 µm‐casting/5% nGel‐KGN. Subsequently, the mineralized core‐shell fibrous layer composed of a PCL or PCL/St. core and a GelA shell were produced directly on the as‐prepared microporous layer through co‐axial electrospinning (Figure 2A). The core solution was prepared by dissolving 10 w/v% PCL into HFIP, where the St. nanowires were homogeneously dispersed. The sheath solution was prepared by dissolving 10 w/v% GelA in HFIP. The co‐axial electrospinning was performed using a co‐axial spinneret consisting of an inner needle (20 G) and an outer needle (14 G). The core and sheath solutions were delivered to the inner and outer needles using two syringe pumps at the flow rate of 0.4 and 0.6 mL h^−1^, respectively, and then electrospun for 10 h at the working distance of 12 cm and the applied voltage of 15–17 kV to produce the core‐shell fibrous layers with the St. concentration of 0 wt.% and 4 wt.%. The fabricated fibrous layers were designated as ES and ES/4% St. After absolutely drying, the fabricated dual‐layer tissue mimetic membranes were crosslinked in EDC/NHS solution (25 mM EDC and 10 mM NHS in absolute ethanol) for 15–20 min. After crosslinking, the membranes were washed with absolute ethanol 3 times, and dried at room temperature. Eventually, three tissue mimetic membranes were prepared by tuning the composition and simply designated as **500C‐ES** (500 µm casting + ES), **500C/nG‐ES/St** (500 µm casting/5% nGel‐KGN + ES/4% St.), and **1000C/nG‐ES/St** (1000 µm casting/5% nGel‐KGN + ES/4% St.).

### Characterization of the Prepared Membranes

The fabricated membranes were cut into small pieces, mounted onto a sample holder, and then coated with Au nanoparticles using a sputter coater (Agar Scientific, UK) at 8 mA for 30 s. Subsequently, the surface morphologies of the samples were examined by SEM (Quanta‐400, FEI, USA) at the accelerating voltage of 10 kV. The cross‐sectional surface of three tissue mimetic membranes was obtained by cutting the samples in liquid nitrogen after being immersed for 5 min and the cross‐sectional morphology was then observed by SEM under the same operation conditions.

TEM (Tecnai G2 Spirit Bio, FEI, USA) analysis was conducted at 120 kV to examine the internal structure and the elemental composition of the polymer–ceramic composite fibers. For the preparation of TEM samples, the core‐shell fibers were directly electrospun onto the carbon‐coated copper grid and dried in the air. STEM (Tecnai F20 ST, FEI, USA) followed by EDS was conducted at 200 kV to examine the internal structure and the elemental composition of PCL/St.‐GelA core‐shell fibers.

The nGel‐KGN‐incorporated microporous layers (500 µm‐casting/5% nGel‐KGN and 1000 µm‐casting/5% nGel‐KGN) in 500C/nG‐ES/St and 1000C/nG‐ES/St membranes were imaged using a Nikon Eclipse Ti inverted microscope (Nikon, Japan) with the exciting wavelength of 488 nm at a *Z*‐stack mode with the step size of 1 µm. The *Z*‐scanning started when fluorescent nGel‐KGN particles first appeared and ended when the particles disappeared within the frame. The sequence of images (512 pixels × 512 pixels) was then processed with ImageJ software (version 1.52k) to obtain the 3D reconstructed images. The values of Z‐aspect and sampling were both set as 3.0 during 3D construction.

The thickness of different layers and three types of tissue mimetic membranes was measured using a thickness gauge (SMD‐565J‐L, Tecklock, Japan) with micrometer accuracy. The chemical structure of the tissue mimetic membranes was identified by attenuated total reflection‐Fourier transform infrared (ATR–FTIR) spectroscopy (Bruker, Germany) in the range of 4000–600 cm^−1^. Thermal analysis of the tissue mimetic membranes was performed using a thermogravimetric analyzer (TGA‐6, Perkin Elmer, USA). The membrane samples (∼10 mg) were heated from 30 to 700 °C at a rate of 10 °C min^−1^ under a nitrogen flow rate of 20 mL min^−1^.

To identify the asymmetric surface topography of the membranes, atomic force microscopy (AFM, NanoWizard 4 XP BioScience, Bruker, USA) was adopted to analyze the surface roughness of the layers at a tapping mode, with the scanning rate of 0.5 Hz and the scanning range of 10 µm × 10 µm. The acquired AFM images were analyzed using the JPK DP data processing software (version 6.0) to obtain the height histogram and root mean square (RMS) roughness of the surfaces.

The surface wettability of the membranes was characterized by the water contact angle (WCA) analysis using an optical video contact angle instrument (OCA25, Dataphysics, Germany) at room temperature. During the WCA measurements, a sequence of images was taken once a water droplet (3 µL) was dropped on the surface, to record the wetting process until the surface was wetted, at the speed of 0.04 s per image. The WCA values at initial and equilibrium states were presented to reveal the wetting process.

### Mechanical Performance of the Prepared Membranes

The mechanical properties of the obtained membranes in a wet state were examined through tensile tests using a material test system (MTS, QTest, Artisan Technology Group, USA), with a 50 N load cell at a crosshead speed of 5 mm min^−1^ at the ambient temperature. All samples were cut into rectangles with dimensions of 10 mm × 50 mm (*n* = 3),^[^ ^18^ ^]^ and then immersed into D. I. H_2_O for 5 min to allow absolute wetting before testing. The thickness of each membrane was measured using a thickness gauge (SMD‐565J‐L, Tecklock, Japan) with micrometer accuracy. The Elastic modulus, tensile strength, and elongation rate were then calculated from the stain–stress curves.

### In Vitro Release and Degradation Behaviors of the Membranes

In the release test, the sterile 500C/nG‐ES/St and 1000C/nG‐ES/St membranes were cut into rectangles with the size of 2 cm × 2 cm, accurately weighed, and then soaked into 5 mL sterile phosphate buffer saline (PBS, 1×, pH 7.40) in 15 mL centrifuge tubes separately (*n* = 3).^[^ ^18^ ^]^ The testing was conducted on a shaker with the rate of 100 rpm, in a 37 °C incubator, for 14 days. At predetermined time intervals, the soaking liquid was partially collected, and replaced by an equal volume of fresh PBS. Concentrations of magnesium ions (Mg^2+^) were determined using an Inductively Coupled Plasma‐Optical Emission Spectrometer (ICP‐OES, ICPE‐9820, Shimadzu, Japan). Concentrations of released nGel‐KGN particles were determined using a fluorescence spectrometer (F‐7000, Hitachi, Japan). The cumulative released concentration of Mg^2+^ and nGel‐KGN particles from a 2 cm × 2 cm membrane was plotted against the time point to obtain the release profile of each membrane. The concentration of Mg^2+^ and nGel‐KGN particles released at the indicated time point was presented as the concentration of Mg^2+^ released from a 2 cm × 2 cm membrane in 5 mL PBS, which was also plotted against time to obtain the release level at the indicated time point of each membrane.

The degradation test was conducted in PBS solutions.^[^ ^72^ ^]^ The sterile membrane was cut into squares with the size of 2 cm × 2 cm, accurately weighed, and then immersed into 5 mL sterile soaking solutions in 15 mL centrifuge tubes separately (*n* = 3). The testing was conducted on a shaker with the rate of 100 rpm, in a 37 °C incubator, for 56 days. At predetermined time intervals, the membrane was taken out, washed 3 times by D. I. H_2_O, completely dried at 37 °C, and then weighed. After weighing, the samples were re‐soaked into 5 mL fresh solution to complete the degradation test. The weight loss ratio (wt.%) at the indicated time point was calculated according to Equation 2, in which *m*_0_ represented the initial weight of the sample, and *m*_1_ denoted the sample weight after degradation for the prescribed period. The weight loss ratio was plotted against time points to gain the degradation profile of each membrane. The data was expressed as the mean ± SD.

(2) $$\mathrm{Weight}\;\mathrm{loss}\;\mathrm{ratio}\;(\mathrm{wt}.\%)=[(m_{0}-m_{1})/m_{0}]\times 100\%$$

After 56‐day degradation in PBS, the surface morphologies of 500C‐ES, 500C/nG‐ES/St, and 1000C/nG‐ES/St membranes were examined by SEM (Quanta‐400, FEI, USA) at the accelerating voltage of 10 kV after sputtered with Au nanoparticles.

### In Vitro Biocompatibility Test

MC3T3‐E1 subclone 14 pre‐osteoblastic cells (ATCC, USA) and NIH3T3 fibroblasts (ATCC, USA) were used for evaluating the In Vitro performance of the obtained membranes. Cells were cultured in α‐MEM (Thermofisher, cat# A10490‐01) supplemented with 10% FBS (Thermofisher, cat# 10270106) and 1% penicillin‐streptomycin (Thermofisher, cat# 15140122) at 37 °C in an atmosphere of 5% CO_2_ and 95% relative humidity. Cells were passaged at ∼80% confluency. For the experiments, cells within 10 passages were used.

The cytotoxicity of the membranes to MC3T3‐E1 and NIH3T3 cells was examined in a direct‐contact way via MTT assay. The MTT test was performed on 3 parallel samples for each membrane. The membranes were cut into squares with the size of 15 mm × 15 mm and sterilized by being immersed in 75% ethanol for 30 min and UV exposure for 2 h. The sterile samples were mounted onto the CellCrown transwells (Sigma, cat# Z742381) and fixed into a 24‐well plate.^[^ ^73^ ^]^ MC3T3‐E1 and NIH3T3 Cells were then seeded onto the membrane separately at the density of 10,000 cells cm^−2^. Cells seeded onto the culture plate were set as the blank control. Plates were then incubated under cell culture conditions. After 3‐day cultivation, the samples were washed with PBS (1×, pH 7.40) to remove any non‐adherent cells. Then, 500 µL culture medium together with 50 µL MTT reagent (5 mg mL^−1^ in PBS, 3‐[4,5‐dimethylthiazol‐2‐yl]‐2,5‐diphenyltetrazolium bromide, Thermo fisher, cat# M6494) was added to the sample. After further incubation for 4 h at 37 °C, 1 mL dimethyl sulfoxide (DMSO, Sigma, cat# D8418) was added to each well to dissolve the formazan crystals. The optical density (OD) was measured using a microplate reader (SpectraMax M3, Molecular Devices Corporation, USA) at a wavelength of 570 nm.

The morphologies of MC3T3‐E1 and NIH3T3 cells were observed via actin and nucleus staining. The MC3T3‐E1 or NIH3T3 cells were seeded on the sterilized samples separately in the same way as described in the cell viability test. Cells seeded onto the culture plate were set as the blank control. After 3‐day cultivation, the samples were rinsed twice with PBS (1×, pH 7.40) to remove any non‐adherent cells and impurities, and then fixed with 3.7% formaldehyde (Sigma, cat# 47608) in PBS (1×, pH 7.40) for 10 min. After fixation, the samples were washed twice and then permeabilized with 0.1% Triton X‐100 (Sigma, cat# T8787) for 5 min. Cellular actin was stained with Alexa Fluor 546 phalloidin (Thermofisher, cat# A22283) for 20 min, and the nucleus was subsequently stained with DAPI (Thermofisher, cat# D1306) for 5 min. After removing the free dyes, the samples were imaged with a Nikon Eclipse Ti inverted microscope (Nikon, Japan) at the exciting wavelengths of 543 nm (red, actin) and 408 nm (blue, nucleus). The relative area of actin filaments was quantified based on the fluorescent images using ImageJ software (version 1.52k). To determine the infiltration depth of the cells, the samples were further imaged at a *Z*‐stack mode with a step size of 1 µm. The *Z*‐scanning started when the cells on the membrane first appeared and ended when the cells disappeared within the frame. The sequence of images (512 pixels × 512 pixels) was then processed with ImageJ software (version 1.52k) to obtain the 3D reconstructed images. During 3D construction, the values of Z‐aspect and sampling were set as 4.0 and 3.0, respectively.

The adhesion and attachment behaviors of the preosteoblastic MC3T3‐E1 cells were observed via SEM imaging (Quanta‐400, FEI, USA) based on the established protocol.^[^ ^34^ ^]^ MC3T3‐E1 cells were seeded on the fibrous surfaces of sterilized samples in the same way as described in the cell viability test, at the density of 10,000 cells cm^−2^. On day 3, samples were rinsed with PBS (1×, pH 7.40) to remove any non‐adherent cells or impurities. The cells adhered to the samples were then fixed with 2.5% glutaraldehyde (Sigma, cat# G5882) in PBS (10×, pH 7.40) overnight at 4 °C. After fixation, the samples were washed with PBS (10×, pH 7.40) 3 times to remove the residual crosslinking agent, and then underwent gradient dehydration with 30, 50, 70, 90, 95, and 100 vol.% ethanol. Finally, the samples were soaked in hexamethyldisilazane (HMDS, Sigma, cat# 440191) for 15 min, and subsequently dried in the air overnight. The samples were then imaged through SEM (Quanta‐400, FEI, USA) at 10 kV after sputtering with Au nanoparticles. The pseudocolor was added to the cells in the SEM images using ImageJ software (version 1.52k). Cell spreading areas were then measured based on the SEM images using ImageJ software (version 1.52k). The measurements were performed on 10–12 random cells from each sample.

### Cell Migration Analysis

The cell migration assessment was performed by the scratch‐wound healing assay using NIH3T3 fibroblasts. Cells were seeded in a 6‐well plate at a density of 200,000 cells/well and cultured for 24 h. Briefly, the sample extracts were prepared by immersing the sterilized membrane specimens into the α‐MEM with 1% FBS at the ratio of 2 cm × 2 cm/5 mL for 72 h under 37 °C. When the cells reached 90% confluence, the monolayer cells were wounded by a sterile 200 µL pipette tip. Each well was washed twice by PBS to remove detached cells. Then, the medium was replaced by the sample extracts. Cells cultured in α‐MEM with 1% FBS were set as the control group. Cells were then cultured in the incubator and imaged at 0, 6, and 24 h post‐treatment at the same position using a Nikon Eclipse Ti inverted microscope (Nikon, Japan). The wound areas at different time points were analyzed by ImageJ software (version 1.52k). The cell migration ratio was calculated through Equation 3

(3) $$\mathrm{Cell}\;\mathrm{migration}\;\mathrm{ratio}\;\left(\%\right)=\left(1-A_{t}/A_{0}\right)\times 100\%$$

where *A*_t_ denoted the wound area at the prescribed time point, and *A*_0_ denoted the wound area at 0 h.

### In Vitro Barrier Performance of the Membranes

The barrier function of membranes was evaluated using NIH3T3 fibroblasts. After sterilization, the membrane with the size of 15 mm × 15 mm was mounted on the CellCrown transwell and fixed into a 24‐well culture plate. NIH3T3 cells were carefully seeded on the microporous surface of the membrane at the density of 10,000 cells cm^−2^. The cell culture medium was refreshed every 2 days. After culturing for 5 days, the two sides of the membrane were observed by SEM (Quanta‐400, FEI, USA) after fixation, following the same protocol described in the cell adhesion test.

### Alkaline Phosphatase (ALP) and Alizarin Red S (ARS) Assays

Osteogenic differentiation of rat bone marrow mesenchymal stem cells (rBMSCs) on the prepared membranes was evaluated through alkaline phosphatase (ALP) and Alizarin Red S (ARS) assays. After sterilization, the membrane with the size of 15 mm × 15 mm was mounted on the CellCrown transwell and fixed into a 24‐well culture plate. Cells at passage 2 were seeded on the membranes at the density of 45,000 cells per membrane and cultured for 2 days to allow for optimal attachment. Cells seeded onto the culture plate were set as the blank control. The cell culture medium was then replaced by the osteogenic induction medium (OIM, Dulbecco's modified eagle medium (DMEM, low glucose, Thermofisher, cat# 11885076) containing 10% FBS, 50 µg mL^−1^ ascorbic acid (Sigma, cat# 795437), 10 nM dexamethasone (Sigma, cat# D1756), and 5 mM β‐glycerolphosphate (Sigma, cat# 50020) for further induction.^[^ ^74^ ^]^ The medium was refreshed every 2 days. ALP (Sigma, cat# 86R‐1KT) staining was performed after induction for 6 and 11 days respectively according to the manufacturer's instructions, and the stained samples were then imaged with a digital camera and an optical microscope. The value of ALP activity was measured after induction for 6 and 11 days respectively using the ALP Assay Kit (Beyotime Biotechnology, cat# P0321S) according to the manufacturer's instructions. ALP activity was then normalized to the total protein content in cell lysate which was determined using the Bradford Protein Assay Kit (Beyotime, cat# P0006) according to the manufacturer's instructions. After induction for 6 and 11 days respectively, ARS (Beyotime, cat# C0148S) staining was performed according to the manufacturer's instructions, and the stained samples were then imaged. To quantify the calcium mineralization, the stained samples were soaked into 10 w/v% cetylpyridinium chloride (Sigma, cat# 1104006) for 1 h. The absorbance was subsequently measured using a microplate reader (Epoch 2S, BioTek, USA) at a wavelength of 570 nm. In both ALP and ARS assays, three parallel samples were used for each group.

### Alcian Blue/Nuclear Fast Red Staining Assay

Chondrogenic differentiation of rBMSCs on the prepared membranes was evaluated through alcian blue staining.^[^ ^14^ ^]^ After sterilization, the membrane with the size of 15 mm × 15 mm was mounted on the CellCrown transwell and fixed into a 24‐well culture plate. Cells at passage 2 were seeded on the membranes at the density of 75,000 cells per membrane and cultured for 2 days to allow for optimal attachment. Cells seeded onto the culture plate were set as the blank control. The cell culture medium was then replaced by the chondrogenic induction medium (StemPro, Thermofisher, cat# A1007101) for further induction. The medium was refreshed every 2 days. After induction for 7, 14, and 21 days respectively, Alcian blue staining (Solarbio, cat# IA2460) was performed according to the manufacturer's instructions, and the stained samples were then imaged.

### In Vitro Expression of Fibrocartilage Markers

The chondrogenic differentiation of rBMSCs was further assessed by quantitative real‐time polymerase chain reaction (RT‐qPCR) to detect the expression of fibrocartilage marker, *SRY‐box transcription factor 9 (Sox9)*.^[^ ^42^ ^]^ After sterilization, the squared membrane (35 mm × 35 mm) was mounted on the CellCrown transwell and fixed into a 6‐well culture plate. rBMSCs at passage 2 were seeded on the membranes at the density of 150,000 cells per membrane and cultured for 2 days to allow for optimal attachment. Cells seeded onto the culture plate were set as the blank control. The cell culture medium was replaced by the chondrogenic induction medium (StemPro, Thermofisher, cat# A1007101) for further induction. The medium was refreshed every 2 days. After induction for 7 days, the total RNA was extracted from the cells using TRIzol reagent (Beyotime, cat# R0016) according to the established protocol.^[^ ^75^ ^]^ The extracted RNA was quantified using an ND‐2000 spectrophotometer (NanoDrop Technologies, Wilmington, DE, USA). Equal quantities (800 ng) of total RNAs from each sample were reverse transcribed to cDNA using a cDNA kit (Takara, cat# 634925). cDNA was amplified with TB Green qPCR SuperMix‐UDG (Takara) and specific primer sequences (Table S2, Supporting Information). The RT‐qPCRs were run on a QuantStudio 12K Flex Real‐time PCR system (Life Technologies, Thermo Fisher Scientific, USA). To determine the mRNA level, 2^−△△Ct^ method was used to calculate the relative expression of mRNA normalizing to the housekeeping gene (*Glyceraldehyde 3‐phosphate dehydrogenase, Gapdh*).

### Animals and Surgical Procedures

Twelve‐week‐old male Sprague Dawley rats were used to establish the rotator cuff tear (RCT) model, which was set to assess the regenerative efficacy of the tissue mimetic membranes.^[^ ^13^, ^47^ ^]^ All rats were kept at the Experimental Animal Center at the Prince of Wales Hospital in Hong Kong under a 12‐h light/dark cycle, 17–24 °C ambient temperature, 70% humidity, and received food and water ad libitum.

The animal surgeries were approved by the Animal Experimentation Ethics Committee of the Chinese University of Hong Kong (Ref. No.: 22‐214‐GRF). Rats were randomly allocated into four RCT groups: control group (Ctrl, without membrane implantation), 500C‐ES group, 500C/nG‐ES/St group, and 1000C/nG‐ES/St group. Rats were anesthetized by intraperitoneal injection of 75 mg kg^−1^ ketamine and 10 mg kg^−1^ xylazine. Rats were placed on a warming operating table while respiration and heart rates were monitored. A deltoid‐splitting incision with a length of 1.5–2 cm was made. Then, the acromioclavicular joint was separated to visualize the rotator cuff. The supraspinatus tendon was cut off at the bone insertion of the greater tuberosity. All soft tissues and fibrocartilage at the tendon–bone interface was debrided. Two non‐absorbable 5‐0 sutures were passed through the supraspinatus tendon in Mason–Allen fashion and bone tunnels were drilled using a 25‐gauge needle into the greater tuberosity. The membrane (3 mm × 3 mm, width × length) was inserted between the supraspinatus tendon and bone, with the microporous layer facing the tendon. The wound was subsequently sutured. All procedures were performed under aseptic conditions. Rats were sacrificed by intraperitoneal injection of an overdose of sodium pentobarbital at weeks 4 and 8 post‐surgery.

### Gait Analysis

Gait analysis was performed to evaluate the gait abnormality by the Catwalk XT 9.0 system (Noldus Information Technology, Wageningen, Netherlands) according to the established protocol.^[^ ^24^ ^]^ Each rat was trained to be familiar with the glass walkway where the rat walked ad libitum for paw print picking. Paw prints were automatically recorded when the rat entered the region of interest (ROI). Paw prints were set as left forelimb, right forelimb, left hindlimb, and right hindlimb by the built‐in software. Successful records for each rat included three times of one crossing that allowed a maximum 60% speed variation without any interruption. The paw prints were manually checked in the system to ensure the correctness of the classifications. Gait analysis parameters included the stand, maximal contact area, maximal contact AT, maximal intensity AT, swing, stride length, single stance, and duty cycle.

### Micro‐Computed Tomography (Micro‐CT) Imaging

The humeral joints were scanned by a vivaCT40 imaging system (SCANCO MEDICAL AG, Switzerland) with a resolution of 19 µm per voxel size. The scanner was set at a voltage of 70 kVp and a current of 114 µA. Twenty slices of the humeral subchondral bone around the growth plate were used to reconstruct the 3D image using the built‐in software.^[^ ^76^ ^]^

### Histological Analysis

Rats were euthanized at weeks 4 and 8 post‐surgery. The isolated humeral joints were fixed in 4% paraformaldehyde (PFA, Thermofisher, USA, cat# A11313) for 48 h. Then the joints were decalcified in 12.5% ethylenediaminetetraacetic acid (EDTA, pH 7.4) for 21 days at room temperature. The EDTA solution was changed every 4 days. The joints were embedded in paraffin and sectioned to 5 µm thickness for histological analysis. Sections were stained with hematoxylin and eosin (H&E, Solarbio, China, cat# G1120) to observe the healing progress of tendon–bone interface. Safranin O/Fast green (Sigma, USA, cat# S8884/F7258) and Picro–Sirius Red (Abcam, UK, cat# ab150681) staining were respectively performed for evaluating the fibrocartilaginous interface regeneration according to the established protocols.^[^ ^24^ ^]^

### Characterization of the In Vivo Biocompatibility by SEM

The biocompatibility of the implanted membranes was evaluated by SEM observations. Briefly, the paraffin sections (5 µm thick) were chronologically dewaxed for 10 min by absolute xylene twice and immersed in absolute ethanol for 5 min. After air drying, the sections were mounted onto sample holders, coated with Au nanoparticles using a sputter coater (Agar Scientific, UK) at 8 mA for 30 s, and then imaged with SEM (Quanta‐400, FEI, USA) at the accelerating voltage of 10 kV.^[^ ^17^ ^]^

### Immunofluorescence Staining

For immunofluorescence staining,^[^ ^24^ ^]^ sections were dewaxed with absolute xylene, followed by rehydration in gradient alcohols (100%, 90%, 80%, and 70%) and finally distilled water. Sections were treated with sodium citrate buffer (10 mM sodium citrate, 0.05% Tween 20, pH 6.0) for 30 min at 80 °C. Then the sections were permeabilized with blocking buffer (1% bovine serum albumin (BSA, Sigma, USA, cat# A7906) and 0.1% Triton X‐100 in PBS) for 30 min at room temperature. The samples were co‐incubated with paired primary antibodies, F‐actin (Thermofisher, USA, cat# MA1‐80729, 1:200) and MMP9 (ABclonal, USA, cat# A2095, 1:100), α‐SMA (Thermofisher, USA, cat# MA5‐11547, 1:200) and Collagen III (Abcam, UK, cat# ab7778, 1:200), and COL2A1 (Santa Cruz, cat# M2139, 1:100) and OCN (Thermo Fisher Scientific, USA, cat# PA5‐78871, 1:200), for 3 h at room temperature. Species‐matched secondary antibodies were incubated without light for 1 h at room temperature. Finally, the sections were mounted with DAPI (Thermofisher, USA, cat# P36931). The negative control was incubated with the same secondary antibody but free of the primary antibody. Images were captured by a microscopic imaging system (Leica DM5500; Leica Microsystems, Wetzlar, Germany) and quantified by ImageJ software (version 1.52v).

### Immunohistochemical Staining

For immunohistochemical staining, sections were dewaxed with xylene, followed by rehydration in gradient alcohols, and finally distilled water. After incubation with 3% hydrogen peroxide for 15 min in the dark, sections were treated with sodium citrate buffer for 30 min at 80 °C. Then, the sections were permeabilized with a blocking buffer containing 0.1% Triton X‐100 for 30 min at room temperature. The sections were incubated with the primary antibody, IL‐6 (Abcam, UK, cat# ab9324), IL‐1β (ABclonal, USA, cat# A1112), or TNFα (ABclonal, USA, cat# A11534), for 2 h at room temperature and subsequently replaced incubation with HRP‐labelled secondary antibody (Abcam, UK, cat# ab6721,1:300; Abcam, UK, cat# ab205719, 1:300) for 1 h. DAB (Abcam, UK, cat# AB64238) was used for color rendering and hematoxylin was used for nucleus location.

### Statistical Analysis

Although all treatments and assessments were not blinded, the results were checked by three independent investigators. All data were displayed as mean ± standard deviation (SD) using GraphPad Prism (version 8.2.1). *P *< 0.05 was defined as statistically significant. One‐way ANOVA with *Tukey's post hoc* test was used for multiple group comparisons. Two‐way ANOVA with *Sidak's post hoc* test was used for the longitudinal measures.

## Conflict of Interest

The authors declare no conflict of interest.

## Supporting information



Supporting Information

Supplemental Movie 1

Supplemental Movie 2

## Data Availability

The data that support the findings of this study are available from the corresponding author upon reasonable request.
