# Supplementary material for: Tissue Mimetic Membranes for Healing Augmentation of Tendon–Bone Interface in Rotator Cuff Repair
Source: Adv Mater. 2025 Jan 31;37(10):2407358. doi: 10.1002/adma.202407358 (PMC11899491; doi:10.1002/adma.202407358)
Supplement: Supplementary file 1 — Supporting Information [file ADMA-37-2407358-s001.docx]

Supporting Information

Tissue Mimetic Membranes for Healing Augmentation of Tendon–Bone Interface in Rotator Cuff Repair

Yuwei Zhu,^#^ Bingyang Dai,^#^ Shian Zhang, Jun Liu, Shunxiang Xu, Weiyang Liu, Xin Chen, Haozhi Zhang, Quan Li, Florence Ou-Suet Pang, Weiguo Li, Chunyi Wen, Ling Qin,* Jiankun Xu,* and To Ngai*

Dr. Y. W. Zhu, Prof. T. Ngai

Department of Chemistry, The Chinese University of Hong Kong, Shatin, N. T., Hong Kong
E-mail: [tongai@cuhk.edu.hk](mailto:tongai@cuhk.edu.hk)

Prof. B. Y. Dai, Mr. S. A. Zhang, Dr. S. X. Xu, Mr. W. Y. Liu, Dr. X. Chen, Dr. H. Z. Zhang, Dr. F. O. Pang, Prof. L. Qin, Prof. J. K. Xu
Musculoskeletal Research Laboratory, Department of Orthopaedics and Traumatology, and Innovative Orthopaedic Biomaterial and Drug Translational Research Laboratory of Li Ka Shing Institute of Health, Faculty of Medicine, The Chinese University of Hong Kong, Shatin, N. T., Hong Kong

E-mail: [jiankunxu@cuhk.edu.hk](mailto:jiankunxu@cuhk.edu.hk), [qin@ort.cuhk.edu.hk](mailto:qin@ort.cuhk.edu.hk)

Prof. Q. Li

Department of Physics, The Chinese University of Hong Kong, Shatin, N. T., Hong Kong

Prof. B. Y. Dai, Mr. J. Liu, Prof. C. Y. Wen

Department of Biomedical Engineering, The Hong Kong Polytechnic University, Hong Kong

Prof. B. Y. Dai

The Hong Kong Polytechnic University Shenzhen Research Institute, Shenzhen, China

Dr. W. G. Li

Department of Orthopaedic and Traumatology, United Christian Hospital, Kwun Tong, Kowloon, Hong Kong

**Supplementary figure and table:**


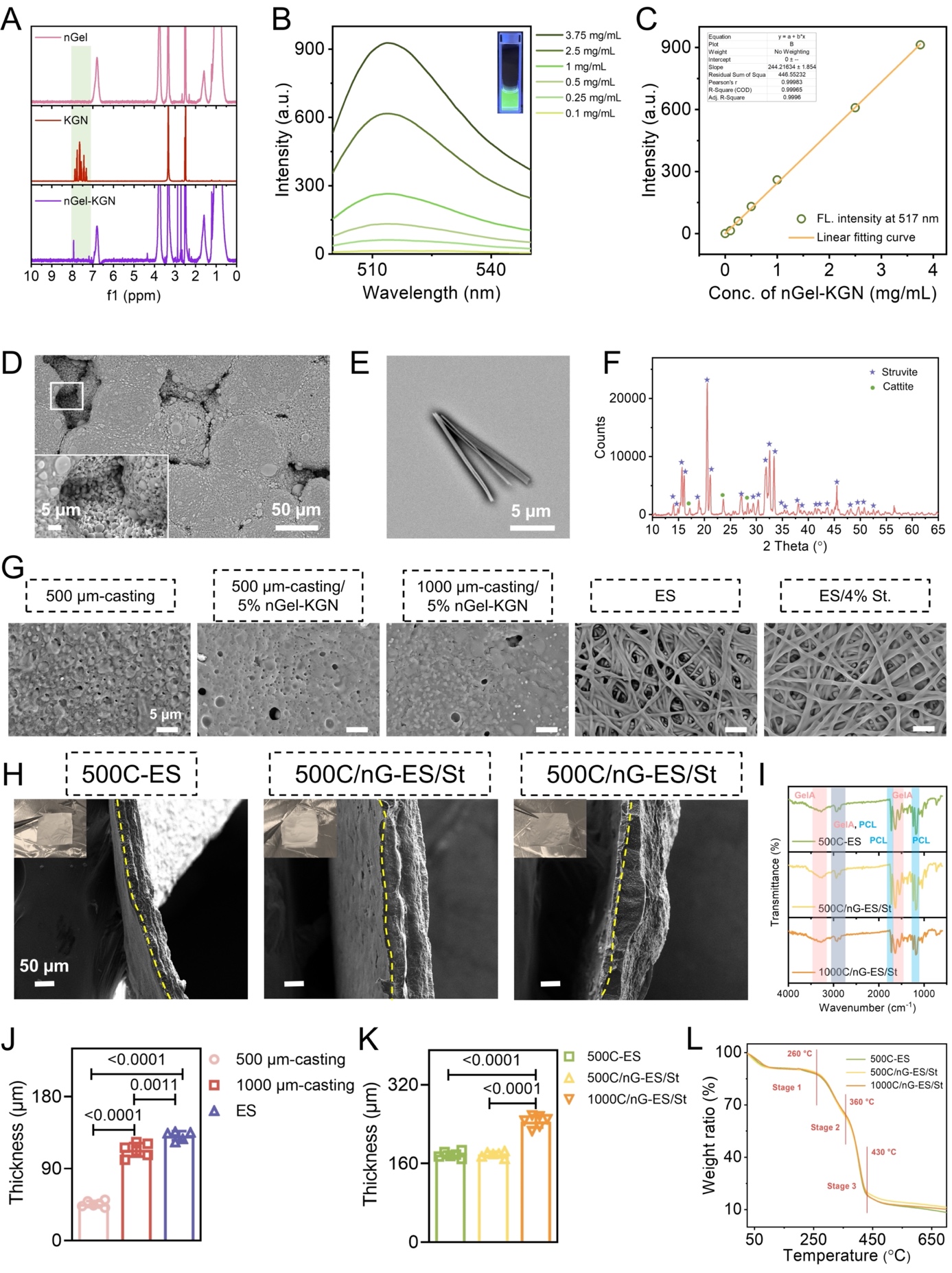


**Figure S1. Additional characterizations of the synthesized nGel-KGN particles, struvite nanowires, and the three types of tissue mimetic membranes (500C-ES, 500C/nG-ES/St, and 1000C/nG-ES/St).** (A) ^1^H NMR spectra of KGN, nGel, and nGel-KGN in deuterated DMSO. (B) Fluorescent spectra of the nGel-KGN dispersions in PBS solution at different concentrations and (C) the corresponding linear fitting curve of the fluorescent emission at 517 nm versus nGel-KGN particle concentration. (D) Surface morphology of the 1000 µm-casting/5% nGel-KGN layer before crosslinking. (E) SEM images of the synthesized struvite nanowires. (F) XRD pattern of the synthesized nanowires. (G) Surface morphologies of the fabricated microporous and fibrous layers: 500 µm-casting, 500 µm-casting/5% nGel-KGN, 1000 µm-casting/5% nGel-KGN, ES, and ES/4% St. (I) ATR-FTIR spectra of the fabricated tissue mimetic membranes. The characteristic peaks are respectively attributed to PCL (in blue) and GelA (in red), and the common peaks of PCL and GelA are highlighted in grey. (J) Thickness of 500 µm-casting microporous layer, 1000 µm-casting microporous layer, and ES layer (*n* = 5). (K) Thickness of the three tissue mimetic membranes (*n* = 6). (L) Thermogravimetric analysis (TGA) curves of the three types of tissue mimetic membranes. The quantitative data are presented as the mean ± SD. One-way ANOVA with *Tukey’s post hoc* test (J, K) was used.


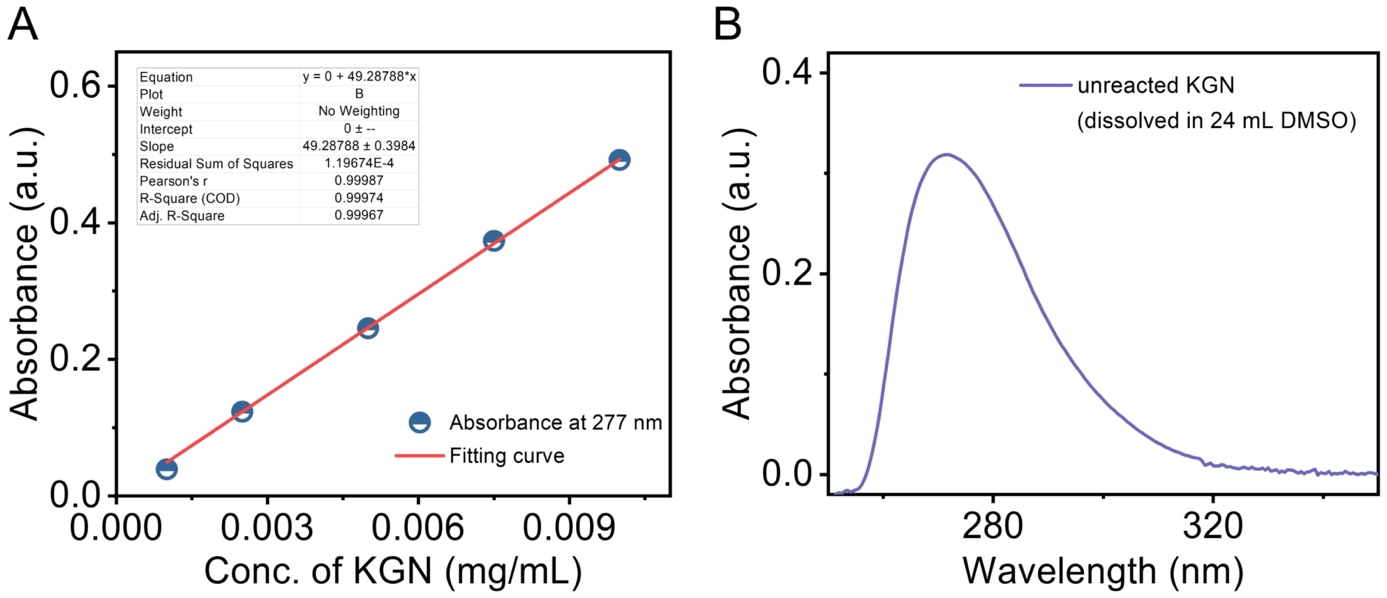


**Figure S2.** **Determination of the binding degree of KGN molecules onto the nGel surface.** (A) Standard curve of the absorbance at 277 nm versus the concentration of KGN standard solution. (B) The UV spectrum of the KGN in 24 mL DMSO, and the absorbance at 277 nm is 0.29643.


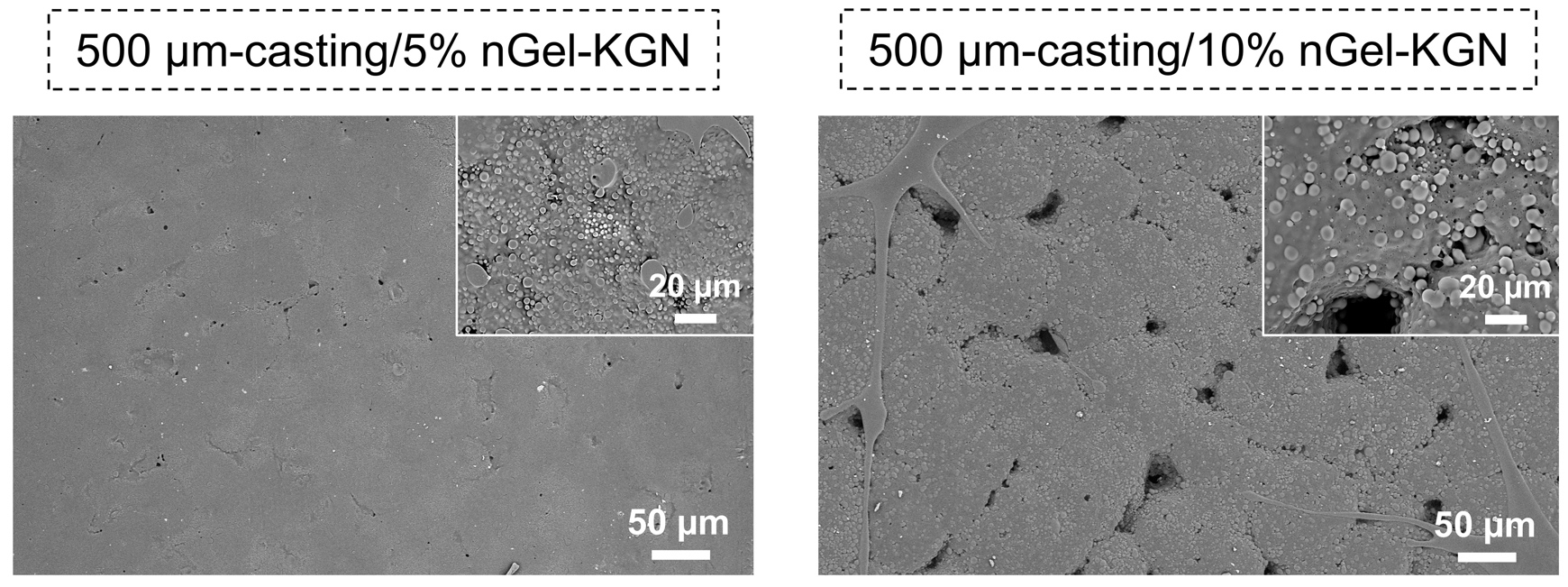


**Figure S3.** Surface morphologies of the microporous layer laden with 5% and 10% nGel-KGN.


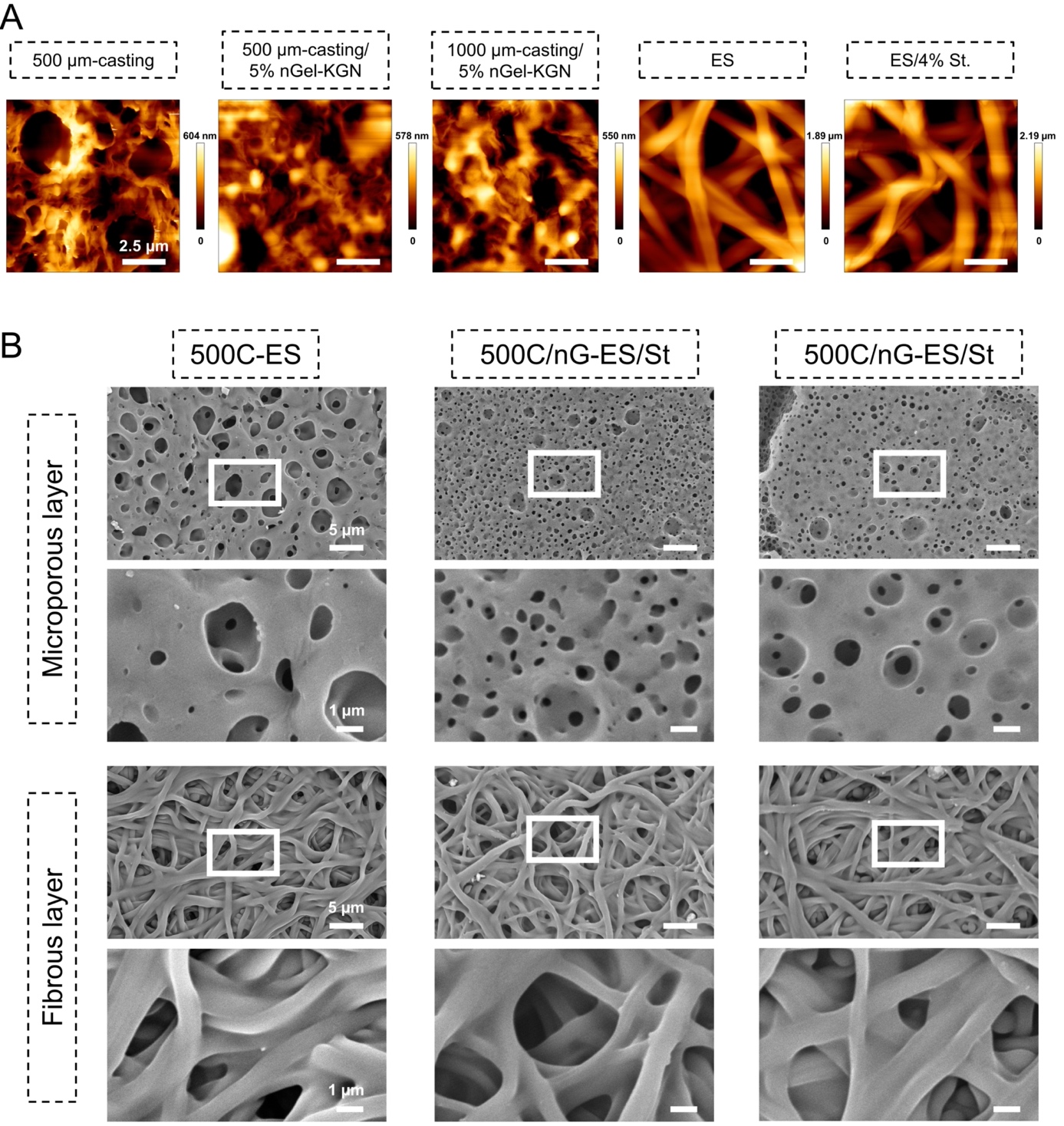


**Figure S4.** (A) 2D AFM images of different microporous and fibrous layers: 500 µm-casting, 500 µm-casting/5% nGel-KGN, 1000 µm-casting/5% nGel-KGN, ES, ES/4% St. (B) Surface morphologies of the three tissue mimetic membranes after 56-day degradation in PBS solution.


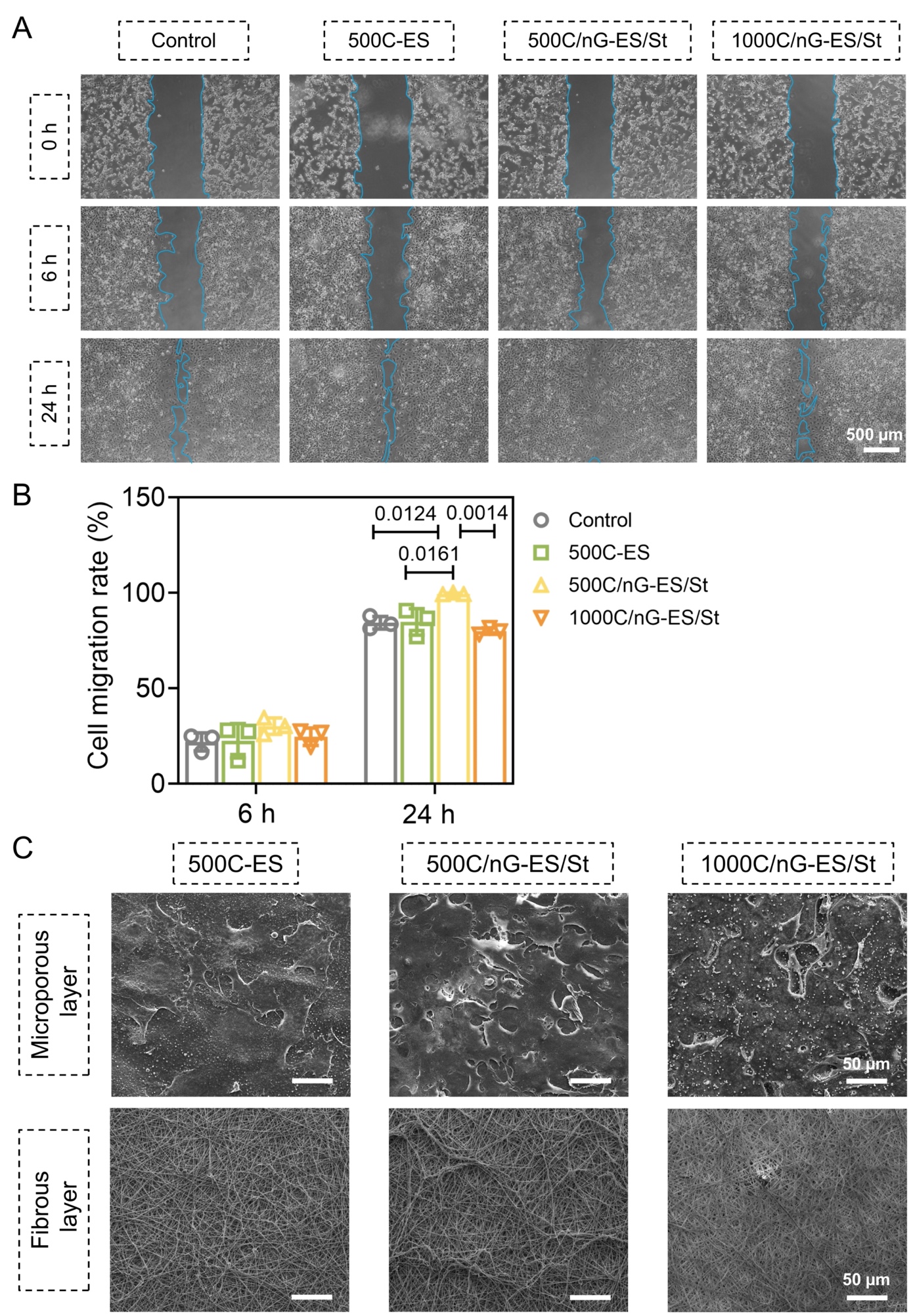


**Figure S5.** (A) Cell migration detected by the scratch-wound healing assay. (B) Corresponding quantification of the cell migration rate (*n* = 3). (C) Representative SEM images of the surfaces of the microporous and fibrous layer in each membrane after the 5-day culture of NIH3T3 cells on the microporous surfaces of the membranes. All quantitative data are presented as the mean ± SD. Two-way ANOVA with *Sidak’s post hoc* test (B) was used.


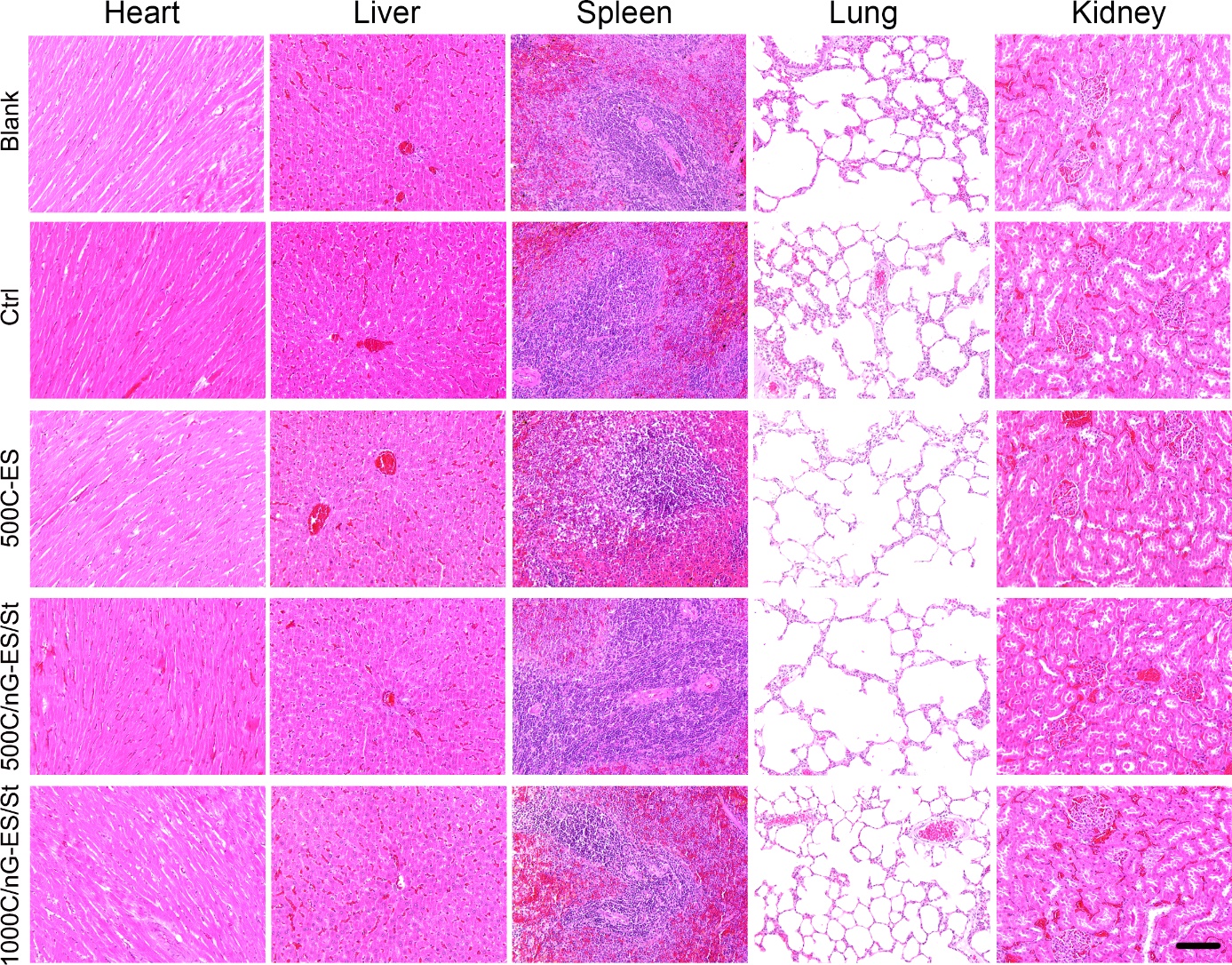


**Figure S6.** H&E staining of visceral organs in rats at week 8 post-surgery. Scale bar: 100 µm.


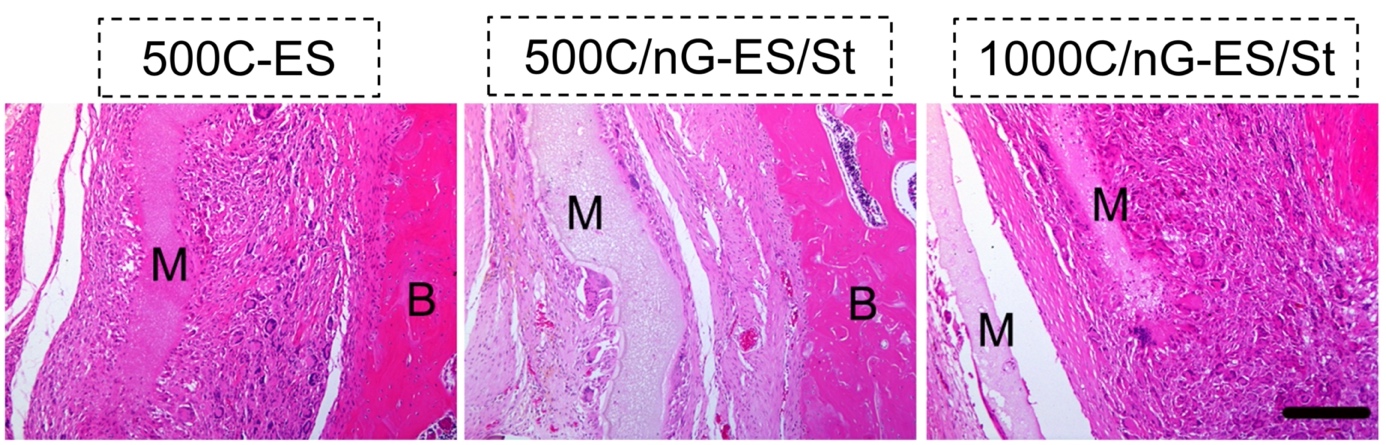


**Figure S7.** H&E staining of implanted membranes at week 8 post-surgery. M: membrane; B: bone. Scale bar: 200 µm.

**Table S1.** Comparison of the three tissue mimetic membranes.

|  | Presence of nGel-KGN and struvite | Thickness of microporous layer | Amount of nGel-KGN |
| --- | --- | --- | --- |
| 500C-ES | **×** | 45.7 ± 3.8 µm | 0 |
| 500C/nG-ES/St | **🗸** | 45.7 ± 3.8 µm | 5% × *m* _(_**_~45.7 µm_** _microporous layer)_ |
| 1000C/nG-ES/St | **🗸** | 113.3 ± 8.4 µm | 5% × *m* _(_**_~113.3_** **_µm_** _microporous layer)_ |

*Note: m* denotes the weight of the microporous layer with different thickness.

**Table S2.** Primer sequences used for RT-qPCR.

| **Gene** | **Oligonucleotide primers** |
| --- | --- |
| *Sox9*- F | TGCTGAGATTCCAGGAGAGAGA |
| *Sox9* - R | AACTCTGAAGGAGCCAAGCC |
| *Runx2* - F | AGCAGACCGTCAAAGGTGTT |
| *Runx2 -* R | GACCCAGAGTCCACCTCTCT |
| *Gapdh* - F | TCTCTGCTCCTCCCTGTTCT |
| *Gapdh* - R | GTTCACACCGACCTTCACCA |

**Supplementary movie:**

**Movie S1.** NTA analysis of the nGel-KGN particles suspended in HEPES buffer at 25 °C.

**Movie S2.** NTA analysis of the nGel-KGN particles suspended in HEPES buffer at 37 °C.
